# RrMYB5‐ and RrMYB10‐regulated flavonoid biosynthesis plays a pivotal role in feedback loop responding to wounding and oxidation in *Rosa rugosa*

**DOI:** 10.1111/pbi.13123

**Published:** 2019-04-14

**Authors:** Yuxiao Shen, Tingting Sun, Qi Pan, Nachaisin Anupol, Hai Chen, Jiewei Shi, Fang Liu, Duanmu Deqiang, Changquan Wang, Jian Zhao, Shuhua Yang, Caiyun Wang, Jihong Liu, Manzhu Bao, Guogui Ning

**Affiliations:** ^1^ Key laboratory of Horticultural Plant Biology Ministry of Education College of Horticulture and Forestry Sciences Huazhong Agricultural University Wuhan China; ^2^ State Key Laboratory of Agricultural Microbiology Huazhong Agricultural University Wuhan China; ^3^ College of Horticulture Nanjing Agricultural University Nanjing Jiangsu China; ^4^ State Key Laboratory of Tea Plant Biology and Utilization College of Tea and Food Science and Technology Anhui Agricultural University Hefei China; ^5^ National Flowers Improvement Center of China Institute of Vegetables and Flowers Chinese Academy of Agricultural Sciences Beijing China

**Keywords:** MYB transcription factor, wounding, oxidation, flavonoid, *Rosa rugosa*

## Abstract

Flavonoids play critical roles in plant responses to various stresses. Few studies have been reported on what the mechanism of activating flavonoid biosynthesis in plant responses to wounding and oxidation is. In this study, flavonoid metabolites and many MYB transcript factors from *Rosa rugosa* were verified to be induced by wounding and oxidation. RrMYB5 and RrMYB10, which belong to PA1‐ and TT2‐type MYB TFs, respectively, showed extremely high induction. Overexpression of *RrMYB5* and *RrMYB10* resulted in an increased accumulation of proanthocyanidins in *R. rugosa* and tobacco by promoting the expression of flavonoid structural genes. Transcriptomic analysis of the transgenic plants showed that most genes, involved in wounding and oxidation response and ABA signalling modulation, were up‐regulated by the overexpression of *RrMYB10*, which was very much similar to that observed in *RrANR* and *RrDFR* overexpression transgenics. RrMYB5 and RrMYB10 physically interacted and mutually activated each other's expressions. They solely or synergistically activated the different sets of flavonoid pathway genes in a bHLH TF EGL3‐independent manner. Eventually, the accumulation of proanthocyanidins enhanced plant tolerance to wounding and oxidative stresses. Therefore, *RrMYB5* and *RrMYB10* regulated flavonoid synthesis in feedback loop responding to wounding and oxidation in *R. rugosa*. Our study provides new insights into the regulatory mechanisms of flavonoid biosynthesis by MYB TFs and their essential physiological functions in plant responses to wounding and oxidative stresses.

## Introduction

Flavonoids play a critical role in plant tissue pigmentation, communicate with environmental cues, such as pollination, and act as antioxidants or signalling molecules to protect plants against ambient environmental challenges (Koes *et al*., 2005). The biosynthetic pathways of anthocyanins and proanthocyanidins (PAs) have been well established. The biosynthesis of flavonoids is regulated by various internal factors (such as biosynthetic enzymes, hormones, transcription factors) and environmental factors (such as temperature, nutrients; Xu *et al*., 2015). The anthocyanin and PA biosynthetic genes at early steps, including the enzymes CHS (chalcone synthase), CHI (chalcone isomerase), F3H (flavanone 3‐hydroxylase), F3′H (flavonoid 3′‐hydroxylase) and F3′5′H (flavonoid 3′,5′‐hydroxylase) and others, are responsible for producing common precursors (Zhao, 2015). Then, the later‐step biosynthetic enzymes [such as DFR (dihydroflavonol‐4‐reductase), FLS (flavonol synthase), LAR (leucoanthocyanidin reductase), LDOX (leucoanthocyanidin dioxygenase), ANR (anthocyanidin reductase) and UFGT (UDP‐glucose: flavonoid 3‐O‐glucosyltransferase)] catalyse the formation of specific end products such as anthocyanins, isoflavones, flavonols and PAs. DFR and LODX catalyse the biosynthesis of three types of dihydroflavonols and their conversion into anthocyanidins. Glycosylation of anthocyanidins is then catalysed by the enzyme UFGT to produce colourful anthocyanins. PAs are synthesized through the conversion of leucocyanidin and anthocyanidins into catechin and epicatechin by the enzymatic activity of LAR and ANR, respectively, before the two monomeric flaval‐3‐ols are polymerized into PA polymers (Xu *et al*., 2015). It has been previously reported that pathogen infections and wounding up‐regulated the PA‐related genes and triggered the accumulation of PAs to reduce damages (Mellway *et al*., 2009). It has also been reported that pap1‐D/fls1 double knockout mutant plants accumulated substantial amount of anthocyanins and exhibited the enhanced tolerance to osmotic stress (Lee *et al*., 2016). Other studies have shown that PAs increased alternative oxidase activity and abscisic acid levels, and finally alleviated stress‐induced oxidative damages in plant cells (Luo *et al*., 2016). However, little is known about how PA feedback affects plant responses to wounding and oxidation and how flavonoid‐regulation‐related TFs are involved in the plant response to wounding and oxidative stresses.

R2R3‐MYB TFs are characterized by multiple functions in regulating various aspects of biological processes. Several MYB TFs are involved in regulating responses to abiotic stresses including wounding (James *et al*., 2017). In *Arabidopsis*,* AtMYB41* and *AtMYB102* were involved in plant resistance against wounding and osmotic stresses (Denekamp and Smeekens, 2003; Lippold *et al*., 2009). Moreover, *AtMYB102* in *A. thaliana* was found in response to feeding by larvae of the white cabbage butterfly *Pieris rapae* (Vos *et al*., 2006). Flavonoid biosynthesis is mainly regulated at the transcriptional level by the MBW (MYB–bHLH–WD40) complex (Jaakola, 2013; Xu *et al*., 2015). The majority of R2R3‐MYBs act as activators to directly or indirectly activate the expression of structural genes of flavonoid biosynthesis pathways (Schaart *et al*., 2013). bHLH TFs usually serve as bridges between MYB and WD40 proteins and directly bind to the promoters of flavonoid biosynthesis genes to promote anthocyanin and PA biosynthesis (Hichri *et al*., 2010; Li *et al*., 2016). However, few documents have reported that the MYBs regulating flavonoid biosynthesis are induced under the wounding or oxidative stress.

Both TT2 and PA1 types of MYB TFs can activate the expression of PA biosynthesis genes, including *DFR*,* LAR* and *ANR* (An *et al*., 2015; James *et al*., 2017; Tian *et al*., 2017 and Wang *et al*., 2018). *AtTT2 (MYB123)* specifically regulates PA accumulation in the seed coat of *Arabidopsis thaliana* (Nesi *et al*., 2001). TT2‐like MYB TFs, including *VvMYBPA2* from grape (Terrier *et al*., 2008), *FaMYB9* and *FaMYB11* from strawberry (Schaart *et al*., 2013), *MdMYB9* and *MdMYB11* from apple (Gesell *et al*.,^2014^), and *PtMYB134* from poplar (James *et al*., 2017; Mellway *et al*.,^2009^), are reported to regulate PA biosynthesis. *AtMYB5*, another type of MYB TF, is also involved in PA and anthocyanin accumulation and is defined as a PA1‐type MYB TF (Schaart *et al*., 2013). The homologs of *AtMYB5* have also been identified in a few woody species, including *DkMYB4* in *Diospyros kaki* (Akagi *et al*., 2009); *VvMYB5a, VvMYB5b* and *VvMYBPA1* in *Vitis vinifera* (Deluc *et al*., 2008); *PtMYB115* in poplar (James *et al*., 2017); and *MdMYBPA1* in apple (Wang *et al*., 2018). Compared with TT2‐type MYB TFs, few reports have detailed how PA1‐type MYB TFs regulate anthocyanin and PA biosynthesis.

*Rosa rugosa* is a commercially important ornamental plant cultivated worldwide, whose populations suffer from herbivorous insects, powdery mildew and blackspot. These stresses negatively impact *Rosa* plant growth, flower quality and field performance (Xing *et al*., 2014). Wounding stress induces the accumulation of important bioactive compounds in wounded tissues (Ioannidi *et al*., 2009; Torres‐Contreras *et al*., 2018). Understanding the responses to the wounding and oxidation of rose plants are of great importance to maintain the yield of these plants since the fresh flowers are frequently cut in *R. rugosa* gardens. However, up to now, few MYB genes have been characterized in rose species, and no reports have revealed the molecular mechanisms in response to wounding and oxidative stresses.

In this paper, we reported the functions of *RrMYB5* and *RrMYB10* from *R. rugosa*. They were both induced by wounding and oxidative stresses and were found to regulate anthocyanin and PA biosynthesis. Overexpression of *RrMYB5* and *RrMYB10* in *R. rugosa* and tobacco resulted in the increased accumulation of PAs. *RrMYB5* and *RrMYB10* physically interacted and promoted the transcriptions of each other, and they solely or synergistically activated flavonoid‐related genes and promoted PA and anthocyanin biosynthesis in a bHLH TF EGL3‐independent manner. The *RrMYB5‐*,* RrMYB10‐*,* RrANR‐* and *RrDFR‐*transgenic tobacco plants and transgenic *R. rugosa* plants with increased accumulation of PAs and anthocyanins displayed an enhanced tolerance to oxidative stress. Therefore, a model of increasing the abiotic tolerance through a flavonoid‐mediated feedback loop in response to wounding and oxidative stresses was proposed. This study is aimed to provide an insight into the regulatory mechanisms of flavonoid biosynthesis under stresses in plants.

## Results

### Flavonoids accumulated and MYB TFs induced in wounded rose leaves and petals

**Figure 1:**
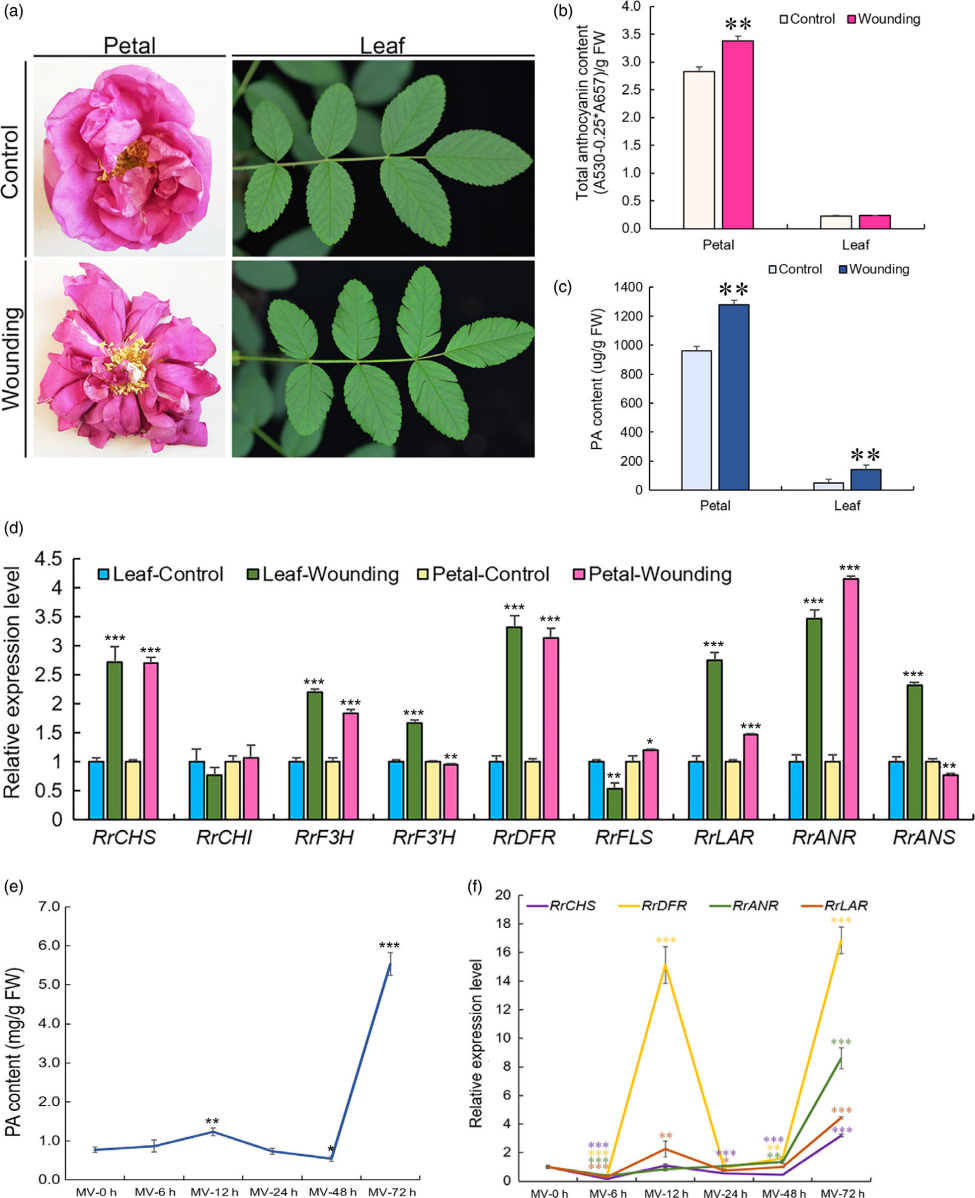
Wounding and oxidation treatments in leaves and petals of *Rosa Rugosa*. (a) Wounding treatment of petals and leaves. (b, c): Total anthocyanins (b); PA (c) contents in wounded leaves and petals. (d) qRT‐PCR analysis of flavonoid‐related gene expression in wounded leaves and petals of *R. Rugosa*. (e) PA content of leaves at different times during the MV treatment. (f) Expression of flavonoid‐related genes in leaves of *R. Rugosa* at different times during the MV treatment. *RrGAPDH* was used as an internal control gene. Data represent mean ± SE of three biological replicates (*n *= 3). Comparison between wounding and control (b, c and d), and MV treatment results at 0 h compared with those at other hours (e and f, respectively). The statistical significance was determined using Student's *t* test (* *P* < 0.05, ** *P* < 0.01, *** *P* < 0.001).

**Figure 2:**
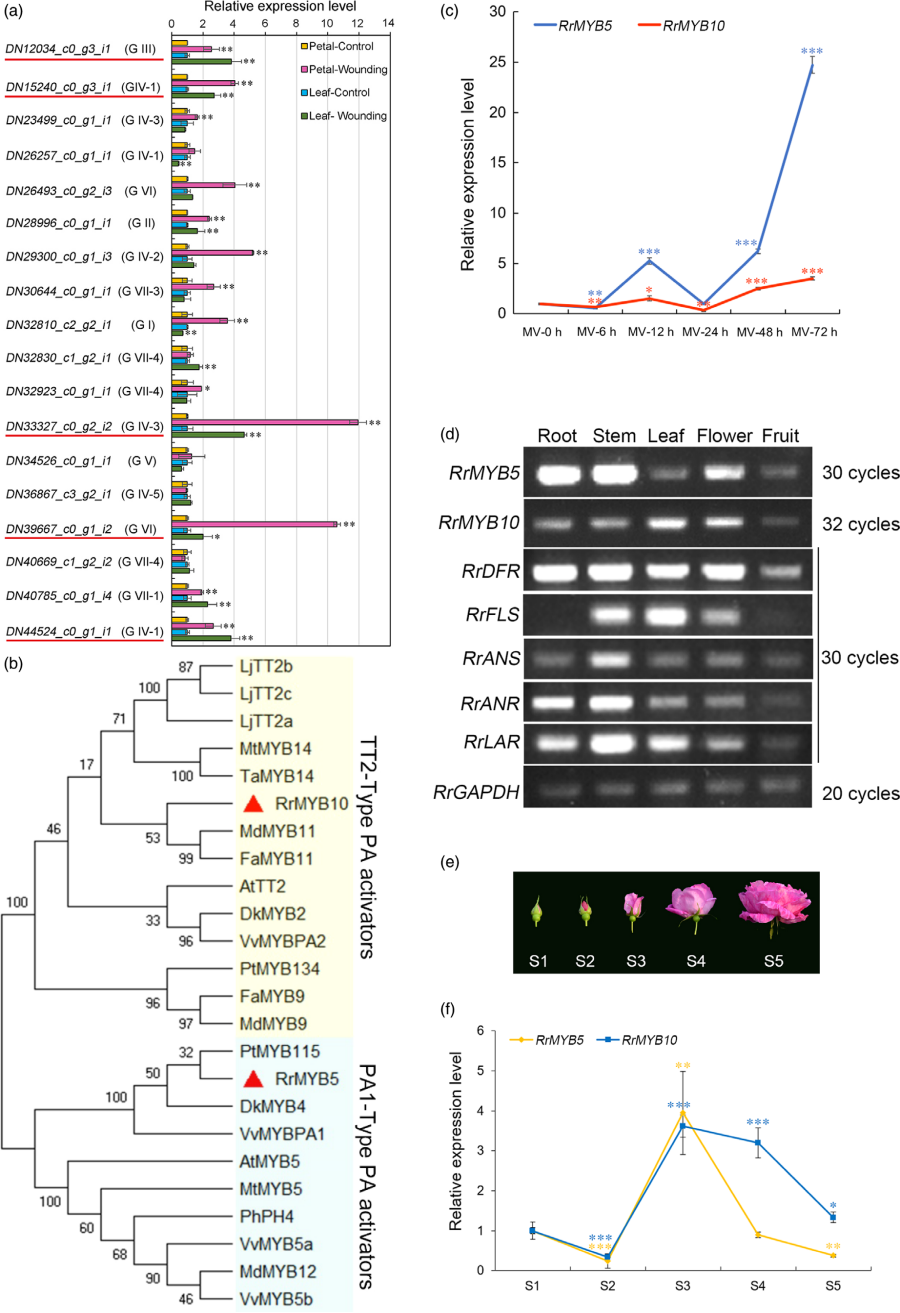
Characterization of selected MYB transcript factors and spatiotemporal expression patterns of *RrMYB5*,* RrMYB10* and flavonoid‐related genes in *R. rugosa*. (a) qRT‐PCR expression profiles of 18 selected MYB genes in wounded petals and leaves. *RrGAPDH* was used as an internal control gene. The red line represents the candidate genes involved in the wounding response. (b) Phylogenetic analysis of PA‐activated MYB genes from different plants. RrMYB5 (DN33327_c0_g2_i1) and RrMYB10 (DN15240_c0_g3_i1) genes are marked by the red triangle. (c) Expression of *RrMYB5* and *RrMYB10* in leaves at different times during the MV treatment. *RrGAPDH* was used as an internal control gene. (d) Semiquantitative RT‐PCR analysis of *RrMYB5*,* RrMYB10* and flavonoid‐related genes in various tissues of *R. rugosa*. (e, f) Stages of *R. rugosa* flowering development (e) and the corresponding expressions of *RrMYB5* and *RrMYB10* (f). *RrGAPDH* was used as an internal control gene, and the flower in S1 stage was used as an internal standard to calculate other samples. Data represent mean ± SE of three biological replicates (*n* = 3). MV treatment results at 0 h were compared with those at other hours (c). (f) Displays the comparison between flower S1 stage and other stages. The statistical significance was determined using Student's t test (* *P* < 0.05, ** *P* < 0.01, *** *P* < 0.001).

Spectrophotometric analysis showed that PAs accumulated in both wounded leaves and petals (Figure 1a) and that anthocyanin only increased in wounded petals (Figure 1b and c). The expression of flavonoid‐related genes (*RrCHS*,* RrF3H*,* RrDFR*,* RrLAR* and *RrANR*) dramatically increased (Figure 1d). qRT‐PCR analysis of the expression of 18 *R. rugosa* MYB genes showed that five MYB genes were significantly up‐regulated (>2‐fold) in both wounded leaves and petals (Figure 2a). These genes were *DN12034_c0_g3_i1*,* DN15240_c0_g3_i1*,* DN33327_c0_g2_i2*,* DN39667_c0_g1_i2* and *DN44524_c0_g1_i1*. A phylogenetic tree was constructed for analysis of the 18 *R. rugosa* MYBs bases on the conserved R2R3‐MYB binding domains (Table S4). The analysis result indicated that these 18 MYBs were clustered into seven groups (Figure S1). DN12034_c0_g3_i1 belonged to group G III and DN39667_c0_g1_i2 belonged to group G VI. DN44524_c0_g1_i1 and DN15240_c0_g3_i1 belonged to subgroup GIV‐1 containing AtMYB123 (AT5G35550) in *Arabidopsis*, which was putatively defined as TT2‐type PA activators. Subgroup GIV‐2 was anthocyanin activators. DN33327_c0_g2_i2 belonged to subgroup GIV‐3, which was PA1‐type PA activators. Subgroup GIV‐4 was flavonol activators. Subgroup GIV‐5 was phenylpropanoid repressors. The three genes (*DN44524_c0_g1_i1, DN15240_c0_g3_i1* and *DN33327_c0_g2_i2*) belonged to the GIV group, and the expressions of the first two genes were up‐regulated more than twofold in wounded petals with the expression of *DN33327_c0_g2_i2* up‐regulated as high as 11.96‐fold. Phylogenetic analysis displayed that all these three genes were homologous to TT2‐type PA activators or PA1‐type PA activators (Figure S1), respectively, with *DN15240_c0_g3_i1* and *DN44524_c0_g1_i1* falling into TT2‐type and the expression of *DN15240_c0_g3_i1* (4.04‐fold) higher than that of DN44524_c0_g1_i1 (2.67‐fold) in wounded petals. Therefore, DN15240_c0_g3_i1 and DN33327_c0_g2_i2 considered to be associated with specific flavonoids were selected for further studies. They were tentatively named RrMYB10 and RrMYB5, respectively.

### Characterization of RrMYB5 and RrMYB10 from *R. rugosa*

*RrMYB5* contained a 858‐bp ORF, encoding 285 aa protein and belonging to the PA1‐type PA activators, and *RrMYB10* contained a 696‐bp ORF, encoding 231 aa protein and belonging to the TT2‐type PA activators, as shown in a phylogenetic tree (Figures 2b and S2). The R2‐R3 MYB domains at N‐termini of RrMYB5 and RrMYB10 were well‐conserved, with high diversity in C‐terminal regions (Figure S2). In addition to being induced by wounding, *RrMYB5* and *RrMYB10* were also induced by oxidation methyl viologen (MV, a strong oxidizer) treatment. PA contents increased over the methyl viologen treatment duration. Twelve hours after treatment, PA levels started to decline gradually and then increased again at 48 h of treatment and attained the highest level at 72 h of treatment (Figure 1e). qRT‐PCR analysis showed that the expression patterns of *RrMYB5* and *RrMYB10* coincided with those of flavonoid structural genes and PA accumulation (Figures 1f and 2c). Therefore, *RrMYB5* and *RrMYB10* might be associated with the PA biosynthesis during the oxidative stress. Motif analysis of the promoters of *RrMYB5* and *RrMYB10* suggested the presence of many motifs related to light and oxidation response (Figure S3A). Under the light treatments, the total anthocyanins accumulated in rose with the increase in expression levels of *RrMYB10*,* RrMYB5* and the structural genes *RrCHS, RrDFR, RrANS, RrANR* and *RrLAR* (Figure S3B‐E).

### Spatiotemporal expression of RrMYB5 and RrMYB10

Semiquantitative RT‐PCR analysis showed that *RrMYB5* was highly expressed in roots, stems and flowers, and that *RrMYB10* was highly expressed in leaves and flowers and exhibited lower transcript levels in the roots, stems and fruits. *RrDFR* was mainly expressed in roots, stems, leaves and flowers. *RrFLS* was mainly expressed in stems and leaves. *RrANS* was highly expressed in stems and next in roots, leaves and flowers. Both *RrANR* and *RrLAR* were highly expressed in stems and roots (Figure 2d). The expression pattern of *RrMYB5* was similar to those of *RrANR* and *RrLAR*. The expression patterns of these genes in flowers at five developmental stages, from unopened bud (stage 1) to fully blooming (stage 5), were examined. The result showed that the transcriptions of both *RrMYB5* and *RrMYB10* peaked at stage 3, after which their transcriptions gradually declined (Figure 2e and f).

### Subcellular location and relationship between RrMYB5 and RrMYB10

**Figure 3:**
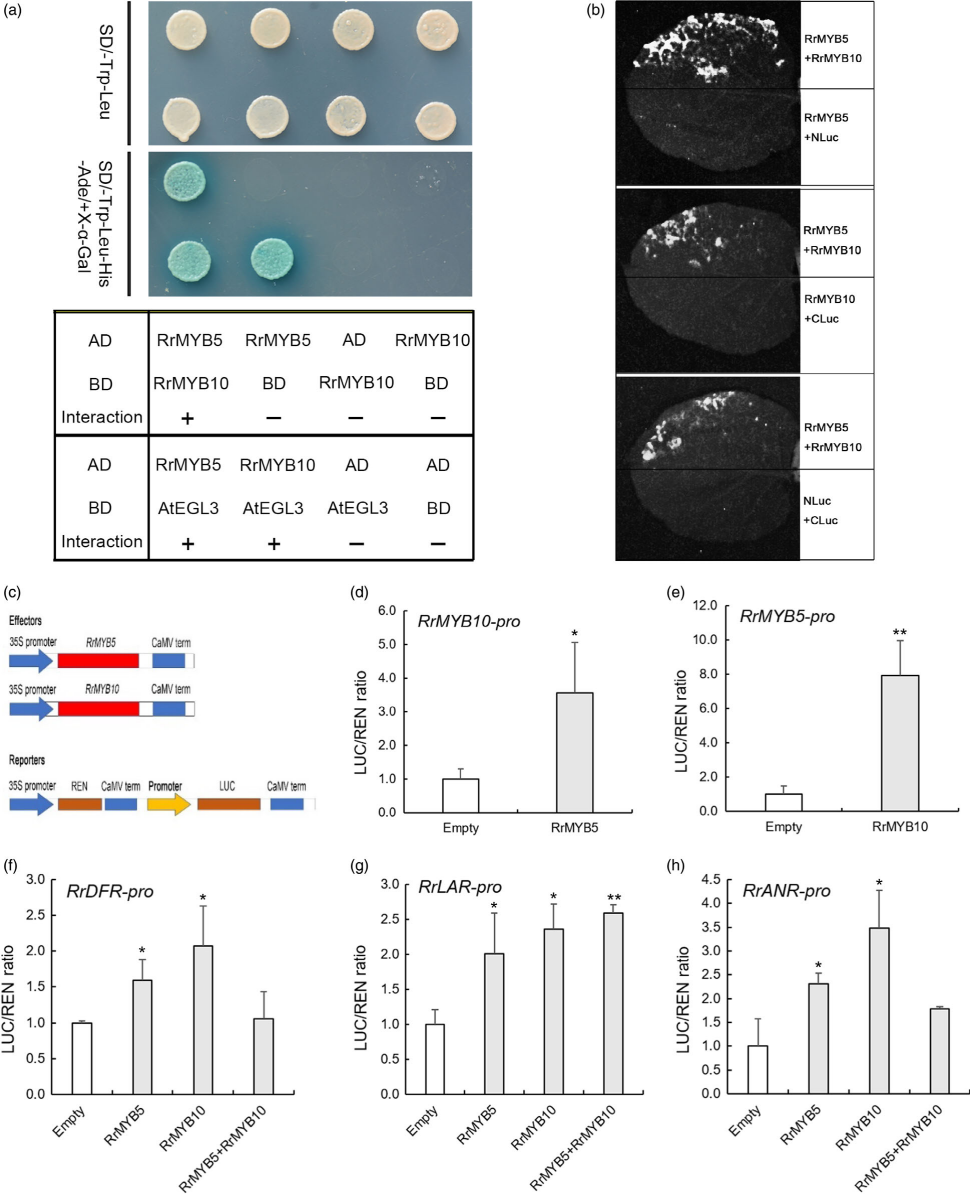
Protein interactions and gene activations through RrMYB5 and RrMYB10. (a) RrMYB5 and RrMYB10 interact with bHLH transcription factor (AtEGL3), and RrMYB5 interacts with RrMYB10 in yeast two‐hybrid assays. (b) RrMYB5 interacts with RrMYB10 in split luciferase complementation assays. The positive luminescence monitored by a CCD camera indicates interaction. (c) Schematic representation of the constructs used for dual‐luciferase assay. The reporter construct contains the firefly luciferase (LUC) driven by the promoter of *RrMYB5, RrMYB10, RrDFR, RrLAR* or *RrANR,* and the Renilla luciferase (REN) driven by the CaMV 35S promoter. The effector constructs contain *RrMYB5* or *RrMYB10* driven by the CaMV 35S promoter. (d, e) *RrMYB5* and *RrMYB10* mutually activate the expression of each other in dual‐luciferase assay. (f–h): *RrMYB5* and *RrMYB10* alone or synergistically activate the expression of *RrDFR* (f), *RrLAR* (g) and *RrANR* (h). The empty pGreenII‐62SK was used as control. The reporters and effectors were coexpressed in *Arabidopsis* protoplast, and both REN and LUC activities were measured. The relative LUC activities normalized to the REN activities are shown (LUC/REN). Data represent mean ± SE of three biological replicates (*n* = 3). The statistical significance was determined using Student's *t* test (* *P* < 0.05; ** *P* < 0.01).

Subcellular localizations of RrMYB5 and RrMYB10 were shown in nuclei (Figure S4). To investigate whether RrMYB5 interacts with RrMYB10, or both of them, respectively, interact with bHLH TF (AtEGL3), yeast two‐hybrid assays were performed. Initially, we found that the RrMYB5 full‐length protein exhibited self‐activation, while RrMYB10 did not (Figures S5b and 3a). Therefore, the C‐termini of the RrMYB5 protein were truncated. The baits carrying the truncated RrMYB5_1–242_, RrMYB5_1–216_ or RrMYB10 fusion protein were co‐transformed with the individual preys harbouring the RrMYB10 or RrMYB5 fusion protein, respectively (Figure S5A). The results showed that both RrMYB5_1‐242_ and RrMYB5_1‐216_ bait interacted with RrMYB10 prey and that RrMYB10 bait interacted with RrMYB5 prey (Figures 3a and S5B). Split luciferase complementation assays in *Nicotiana benthamiana* further confirmed the interaction between RrMYB5 and RrMYB10 (Figures 3b and S5C). In addition, both RrMYB10 and RrMYB5 interacted with AtEGL3, a bHLH TF, to form a transcriptional complex (Figure 3a).

### RrMYB5 and RrMYB10 activate promoters of flavonoid‐biosynthesis‐related genes in transiently transformed Arabidopsis protoplasts

*In vivo* dual‐luciferase reporter assays showed that RrMYB5 and RrMYB10 activated each other, that they also regulated the expression of *DFR*,* LAR* and *ANR* (Figure 3c and d‐h). RrMYB5 interacted with RrMYB10. The obtained RrMYB5‐RrMYB10 complex showed lower trans‐activation activity towards the *RrDFR* and *RrANR’*s promoters than either individual MYB TF did alone (Figure 3f and h). Both RrMYB10 and RrMYB5 interacted with bHLH protein EGL3 and synergistically activated the promoters of *DFR, ANR* and *LAR* (Figure S6A‐E). Therefore, RrMYB5 and RrMYB10 alone or in their interaction complex activated the PA biosynthesis through up‐regulation of PA synthetic genes.

### Overexpression of RrMYB5 and RrMYB10 leads to increased expression of PA biosynthesis genes and enhanced PA production in *R. rugosa*

**Figure 4:**
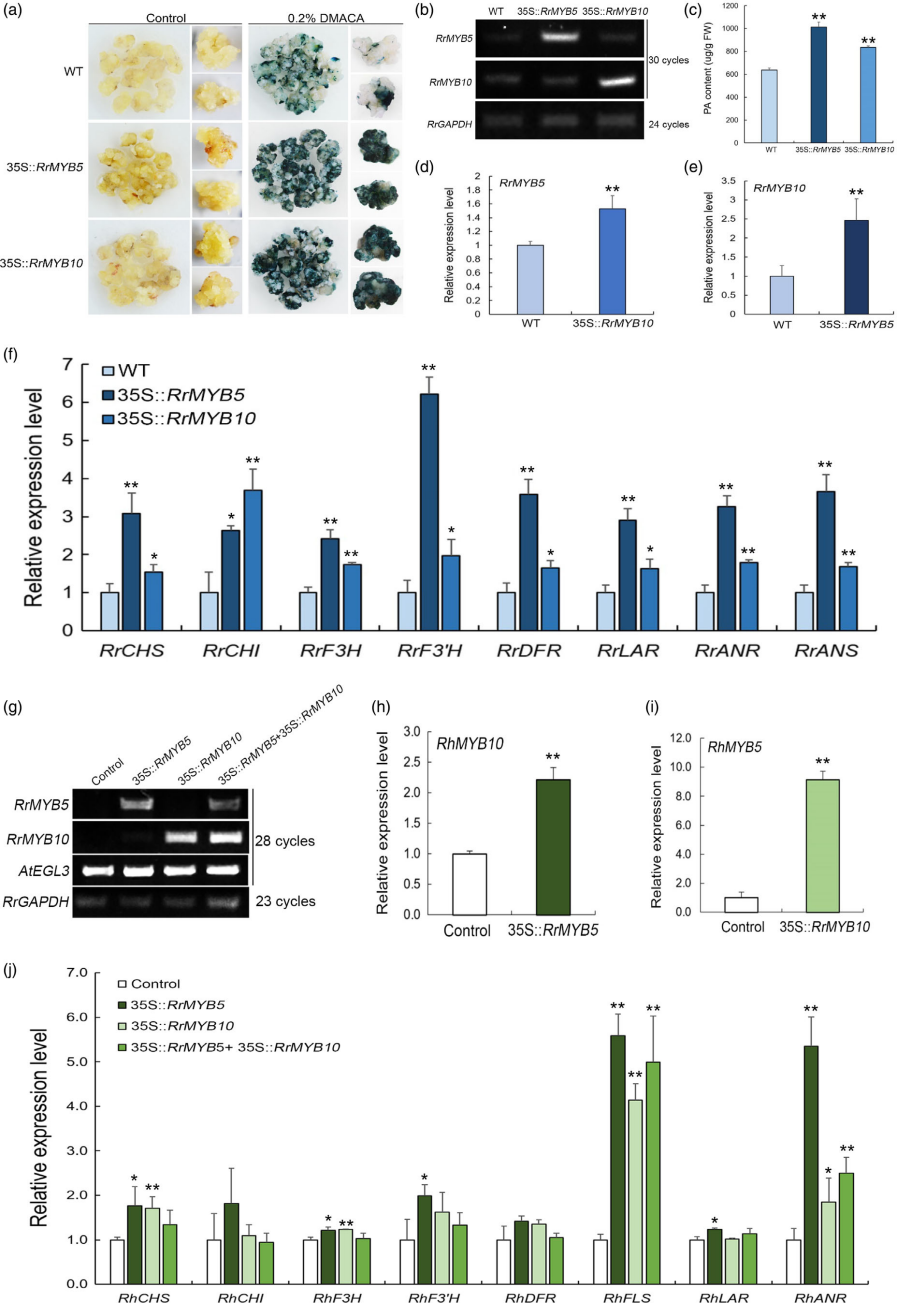
Phenotypes of transgenic *R. rugosa* harbouring *RrMYB5* and *RrMYB10* genes. (a) DMACA staining of the transgenic somatic embryos of *R. rugosa* harbouring 35S::*RrMYB5*, 35S::*RrMYB10* and wild type (WT). (b) Semiquantitative RT‐PCR analysis of *RrMYB5* and *RrMYB10* expression in transgenic embryos. (c) PA contents in transgenic embryos of *R. rugosa*. (D‐E) qRT‐PCR analysis of *RrMYB5* and *RrMYB10* in transgenic embryos. (f) qRT‐PCR analysis of flavonoid‐related genes expression in transgenic *R. rugosa*. (g) Semiquantitative RT‐PCR analysis of *RrMYB5*,* RrMYB10* and *AtEGL3* in transiently transfected rose petals. *RrGAPDH* was used as an internal control gene. (h, i) qRT‐PCR analysis of *RhMYB10* (the homologous gene to *RrMYB10*) and *RhMYB5* (the homologous gene to *RrMYB5*) expression in *RrMYB5* and *RrMYB10* transfected rose petals, respectively. (j) qRT‐PCR analysis of flavonoid‐related gene expression levels in transiently transfected rose petals. Data represent mean ± SE of three biological replicates (*n* = 3). The statistical significance was determined using Student's *t* test. (* *P* < 0.05; ** *P* < 0.01).

To test the function of *RrMYB5* and *RrMYB10*, we genetically introduced 35S::*RrMYB5* and 35S::*RrMYB10*, respectively, into somatic embryos of *R. rugosa* ‘Bao white’. The *RrMYB5‐* and *RrMYB10*‐transgenic embryos showed a yellow colour, and no obvious difference from the wild type (Figure 4a). The transgenic embryos, in which *RrMYB5* or *RrMYB10* was highly expressed (Figure 4b), were stained deep blue, whereas the wild type was stained light blue by DMACA (Figure 4a). PA quantification indicated that *RrMYB5‐* and *RrMYB10‐*overexpressing embryos produced more PAs than the wild type (Figure 4c). Moreover, *RrMYB5* and *RrMYB10* genes were highly expressed in *RrMYB10‐* and *RrMYB5*‐transgenic embryos, respectively (Figure 4d–e). qRT‐PCR analysis of PA biosynthetic genes showed that the expressions of *CHS, CHI, F3H, F3′H, DFR, LAR, ANR* and *ANS* were all significantly increased (Figure 4f).

Since RrMYB5 interacted with RrMYB10 and both RrMYB5 and RrMYB10 interacted with bHLH transcription factor *AtEGL3*, transient expression assays were undertaken in *R. hybrida* petals by using single genes or coexistence‐interacted genes. RT‐PCR confirmed the high expression of target genes in transgenic petals (Figure 4g). qRT‐PCR analysis showed that the expressions of *RhMYB10* and *RhMYB5*, two homologous genes of *RrMYB10* and *RrMYB5* in *R. hybrida*, respectively, were significantly up‐regulated in petals (Figure 4h,i). The expressions of *RhCHS, RhF3H, RhFLS and RhANR* were all significantly increased in the *RrMYB5‐* or *RrMYB10*‐transgenic petals. *RrMYB5* individually up‐regulated the expressions of *RhF3′H* and *RhLAR*. Moreover, those genes had lower expression levels in the presence of both *RrMYB5* and *RrMYB10* than in the presence of either of them. In addition, the expressions of *RhCHI* and *RhDFR* genes showed no differences in all combinations (Figure 4j).

### Ectopic expression of RrMYB5 and RrMYB10 leads to enhanced accumulation of PAs and anthocyanins in tobacco

**Figure 5:**
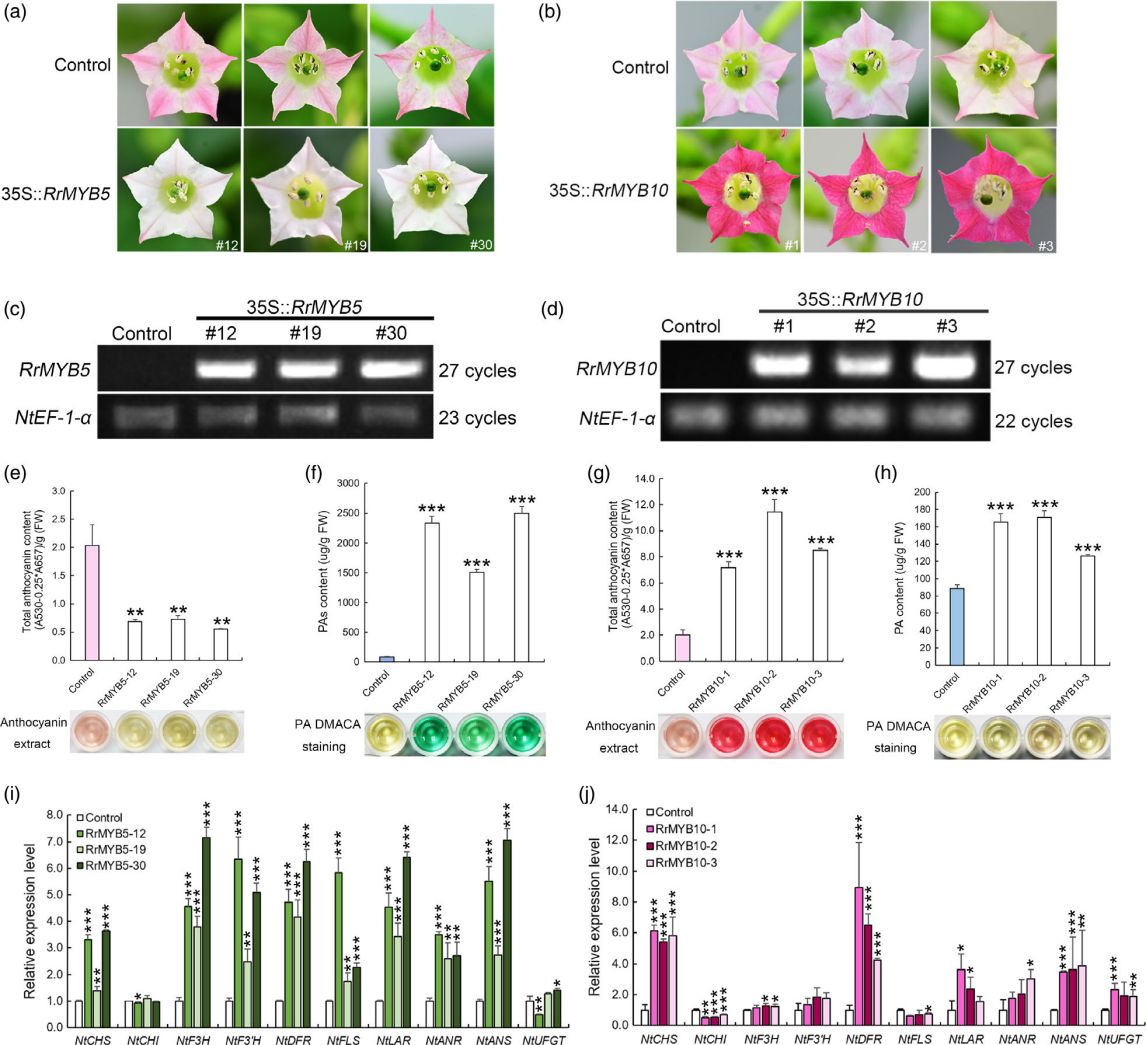
Phenotypes of transgenic tobacco harbouring the *RrMYB5* and *RrMYB10* genes. (a) Pigmentation of flower petals of control and *RrMYB5*‐transgenic tobacco plants. (b) Pigmentation of flower petals of control and *RrMYB10*‐transgenic tobacco plants. (c) Semiquantitative RT‐PCR analysis of *RrMYB5* expression in transgenic lines. (d) Semiquantitative RT‐PCR analysis of *RrMYB10* expression in transgenic lines. (e, f) Total anthocyanin levels and PA levels in the flowers of *RrMYB5*‐transgenic tobacco. (g, h) Total anthocyanin levels and PA levels in the flowers of *RrMYB10‐*transgenic tobacco. (i) qRT‐PCR analysis of flavonoid‐related gene expression in *RrMYB5*‐transgenic tobacco. (j) qRT‐PCR analysis of flavonoid‐related gene expression in *RrMYB10*‐transgenic tobacco. Data represent mean ± SE of three biological replicates (*n* = 3). The statistical significance was determined using Student's *t* test. (* *P* < 0.05; ** *P* < 0.01; *** *P* < 0.001).

In *RrMYB5‐* and *RrMYB10*‐transgenic tobacco, the petals of *RrMYB5‐*overexpressing plants presented white or light pink colour, whereas those of *RrMYB10* overexpressing plants presented red colour (Figure 5a and b). A great difference was observed between the plants overexpressing *RrMYB5* and *RrMYB10* and the control (Figure 5c and d). Quantification assays showed that the PA and anthocyanin contents were significantly increased in *RrMYB10* overexpressing lines (Figure 5g and h), whereas the total anthocyanin contents decreased and PA contents significantly increased in *RrMYB5*‐transgenic tobacco petals (Figure 5e and f). qRT‐PCR assay showed that those genes involved in the flavonoid pathway, including *NtCHS*,* NtF3′H*,* NtDFR*,* NtANS*,* NtANR* and *NtLAR*, were significantly up‐regulated in both *RrMYB5‐* and *RrMYB10*‐transgenic tobacco (Figure 5i and j). Therefore, overexpression of *RrMYB5* and *RrMYB10* can lead to a significantly increased accumulation of PAs in tobacco. The anthocyanins were also significantly accumulated in *RrMYB10*‐transgenic tobacco (Figure 5b and g).

### Transcriptomic profiling of tobacco plants overexpressing RrMYB10, RrDFR and RrANR

RNA‐seq analyses of the *RrMYB10‐* and *RrDFR*‐transgenic tobaccos were integrated with transcriptomic profiling of *RrANR* previously taken by us (Figure S7). The results revealed that 1330 and 1806 differentially expressed genes (DEGs) were up‐ or down‐regulated, respectively, in the *RrMYB10*‐transgenic tobacco, compared with the control (Table S8). A total of 612 and 722 DEGs were up‐ or down‐regulated, respectively, in the *RrDFR*‐transgenic tobacco (Table S9), and 3075 and 3106 DEGs were up‐ or down‐regulated, respectively, in the *RrANR‐*transgenic tobacco (Table S10). These results suggested that overexpression of *RrMYB10*,* RrANR* and *RrDFR* all had a significant impact on global gene expression (Figure S11). Three main categories (‘biological process’, ‘molecular function’ and ‘cellular component’) were classified by GO enrichment analysis. In the ‘biological process’ category, most DEGs were related to the ‘metabolic process’ subcategory, and others were related to ‘cellular process’ and ‘response to stimulus’ (Figures S8–S10). The analyses indicated that *RrMYB10*,* RrDFR* and *RrANR* might be related to the secondary metabolite biosynthesis and the responses to stress stimulation.

**Figure 6:**
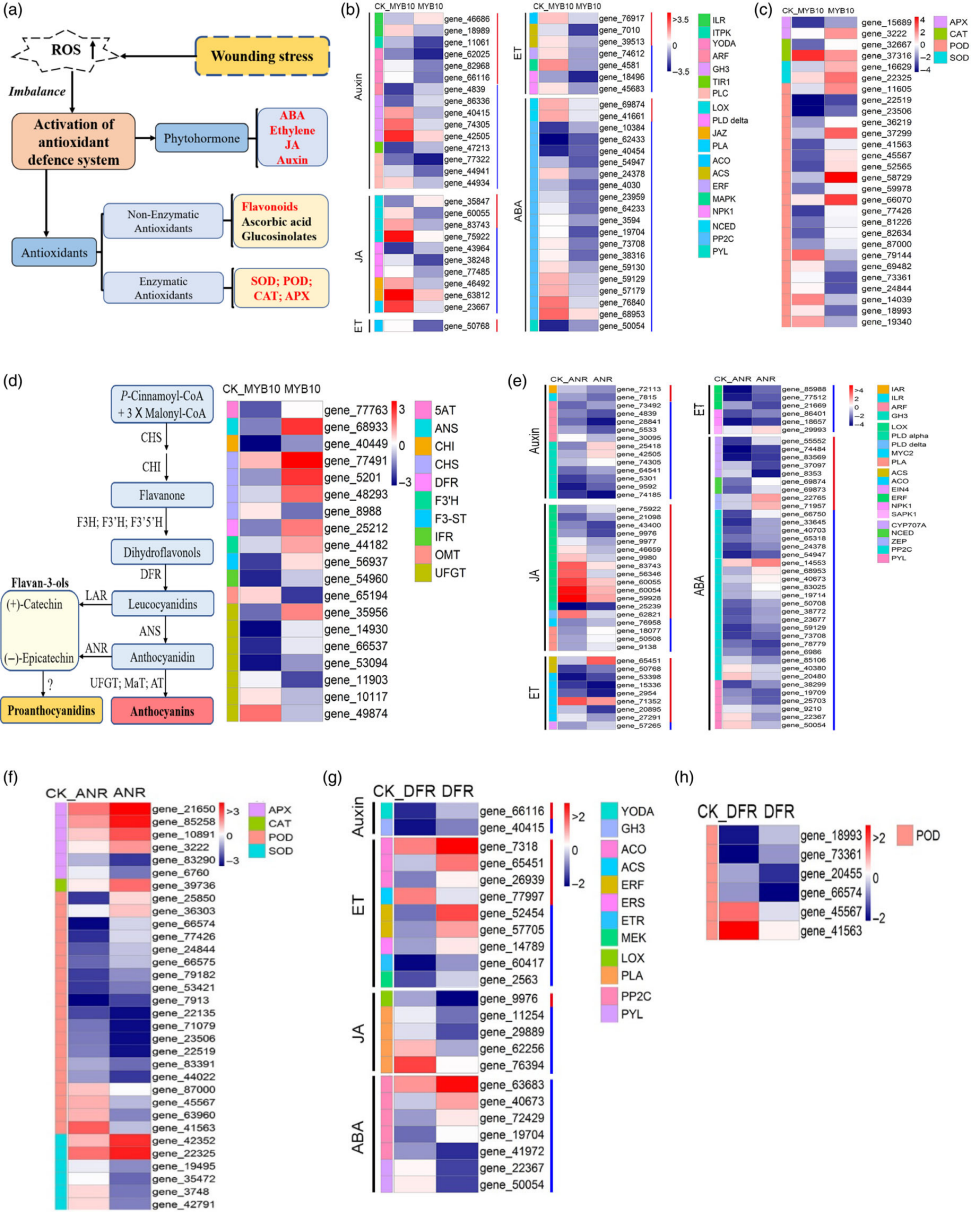
Transcript abundance in flowers from *RrMYB10‐, RrANR‐* and *RrDFR*‐transgenic tobacco plants. (a) Diagram of the antioxidant defence system in plants. (b) Heatmap shows the expression of genes associated with hormone anabolic metabolism and catabolism and signalling pathways in *RrMYB10*‐transgenic tobacco. Log_2_ expression values were calculated with the following formula: log_2_ (values + 1). The expression values are presented relative to the mean expression (*n* = 3). The value of 0 was arbitrarily set to the lowest value of −3.5. (c) Heatmap shows the expression of antioxidant‐related genes in *RrMYB10*‐transgenic tobacco plants. (d) Diagram of the flavonoid biosynthetic pathway (left) and heatmap showing the expression of flavonoid‐related genes in wild‐type (WT) and *RrMYB10*‐transgenic tobacco flowers (right). (e) Heatmap shows the expression of genes associated with hormone anabolic metabolism and catabolism and signalling pathways in *RrANR‐*transgenic tobacco plants. (f) Heatmap shows the expression of antioxidant‐related genes in *RrANR‐*transgenic tobacco plants. (g) Heatmap shows the expression of genes associated with hormone anabolic metabolism and catabolism and signalling pathways in *RrDFR‐*transgenic tobacco plants. (h) Heatmap shows the expression of antioxidant‐related genes in *RrDFR‐*transgenic tobacco plants. Red lines represent the genes involved in hormone anabolic metabolism and catabolism. Blue lines represent the genes involved in hormone signalling transduction pathways. ET, ethylene; JA, jasmonic acid; ABA, abscisic acid; APX, ascorbate peroxidase; CAT, catalase; POD, peroxidase; SOD, superoxide dismutase.

Plants have evolved complicated responsive and adaptive strategies to against harsh and fluctuating environmental conditions (Figure 6a). The analysis of DEGs associated with the anabolic metabolism and catabolism and signalling transduction of plant hormones including auxin, ethylene, jasmonic acid and ABA showed that 53 DEGs and 83 DEGs were significantly changed in *RrMYB10* (Figure 6b)‐ and *RrANR* (Figure 6e)‐transgenic tobacco, respectively, and that 23 DEGs associated with plant hormones were significantly changed in *RrDFR‐*transgenic tobacco (Figure 6g). The expression level of ABA‐related genes, especially ABA signalling genes, showed great changes in *RrMYB10‐* and *RrANR*‐transgenic tobacco (Figure 6b and e). A total of 14 PP2Cs, belonging to ABA signalling gene, were significantly down‐regulated in *RrMYB10*‐transgenic plants, and 5 PP2Cs and 1 PP2Cs were also down‐regulated in *RrANR‐* and *RrDFR*‐transgenic plants, respectively. The above results suggested that ABA‐related genes were significantly influenced in *RrMYB10‐* and *RrANR‐*transgenic plants.

A total of 15 DEGs associated with flavonoid biosynthesis were significantly up‐regulated in *RrMYB10* (Figure 6d). Additionally, a number of DEGs encoding stress‐responsive proteins were up‐regulated in the *RrMYB10*‐, *RrANR*‐ and *RrDFR*‐overexpressing tobaccos. Antioxidant‐related genes were dramatically up‐regulated in transgenic tobacco. A total of 20 DEGs and 16 DEGs annotated as antioxidant‐related genes were significantly up‐regulated in *RrMYB10* (Figure 6c)‐ and *RrANR* (Figure 6f)‐transgenic tobacco, respectively, and 2 DEGs were up‐regulated in *RrDFR‐*transgenic tobacco (Figure 6h). Fourteen common DEGs in *RrMYB10‐* and *RrANR*‐transgenic tobacco (Figure S12A) and four common DEGs in *RrMYB10* and *RrDFR* (Figure S12B), which were associated with plant hormones, were significantly up‐regulated. Nine common DEGs in the *RrMYB10‐* and *RrANR*‐transgenic tobacco (Figure S13A) and four common DEGs in the *RrMYB10* and *RrDFR* (Figure S13B), which encoded ROS scavenging enzymes, were obviously up‐regulated.

### Oxidative stress tolerance in transgenic *R. rugosa*

**Figure 7:**
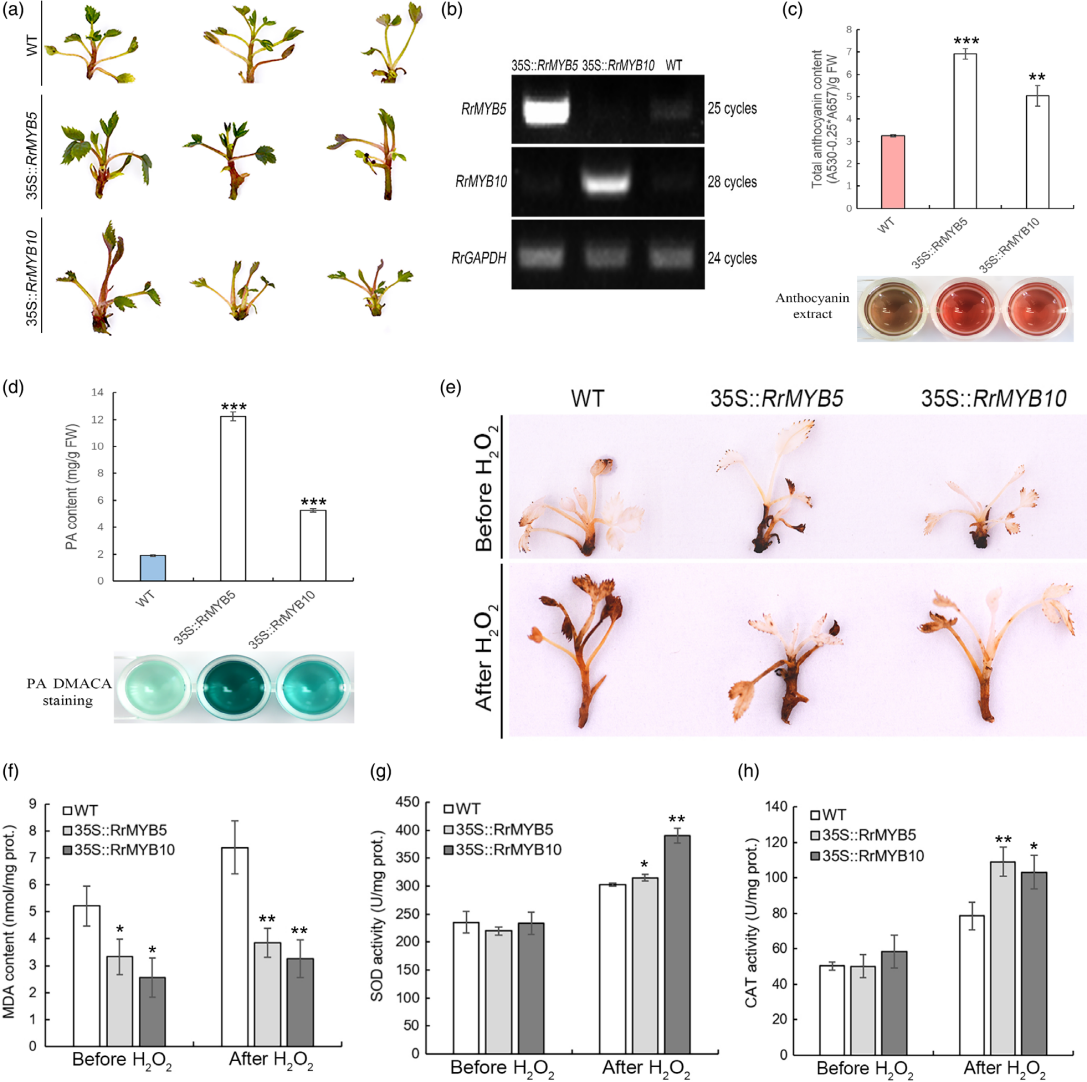
Phenotypes and oxidative stress tolerance of *RrMYB5‐* and *RrMYB10‐*transgenic *R. rugosa*. (a) Phenotype of transgenic *R. rugosa* harbouring the *RrMYB5* and *RrMYB10* genes. (b) Representative semiquantitative RT‐PCR analysis of RrMYB5 and RrMYB10 expression in transgenic *R. rugosa*. (c, d) Total anthocyanin levels (c) and PA levels (d) in shoots of *RrMYB5‐* and *RrMYB10*‐transgenic *R. rugosa*. (e) Representative photographs of *RrMYB5‐* and *RrMYB10*‐transgenic *R. rugosa* showing staining with DAB before and after H_2_O_2_ treatment. (f) MDA levels in *R. rugosa* wild‐type (WT) and transgenic plants before and after H_2_O_2_ treatment. (g and h) SOD (g) and CAT (h) activity in wild‐type (WT) and transgenic *R. rugosa* before and after H_2_O_2_ treatment. Data represent mean ± SE of three biological replicates (*n* = 3). The statistical significance was determined using Student's *t* test (* *P* < 0.05; ** *P* < 0.01; *** *P* < 0.001).

The regenerated shoots of *RrMYB5‐* and *RrMYB10‐*transgenic *R. rugosa* displayed the more obvious pigmentation of anthocyanins than wild type (Figure 7a). The overexpression of *RrMYB5* and *RrMYB10* in all the transgenic plants was verified by semiquantitative RT‐PCR (Figure 7b). Both the anthocyanins and PAs were found to be significantly accumulated in transgenic *R. rugosa* (Figure 7c and d). Before the stress treatment, the DAB staining exhibited no difference between wild‐type (WT) and transgenic rose, while stronger DAB staining was observed in wild type after 30‐h H_2_O_2_ treatment (Figure 7e). Although H_2_O_2_ treatment led to the increase of MDA contents in both transgenic shoots and wild type, MDA contents in the transgenic shoots were significantly lower than those in the wild type (Figure 7f). Before the H_2_O_2_ treatment, no difference in CAT and SOD activities was observed between the wild‐type (WT) and transgenic rose, while after the stress treatment, the *RrMYB5‐* and *RrMYB10*‐transgenic shoots showed the higher levels of SOD and CAT activities than the wild type (Figure 7g, h).

### Oxidative stress tolerance in the four transgenic tobacco plants

**Figure 8:**
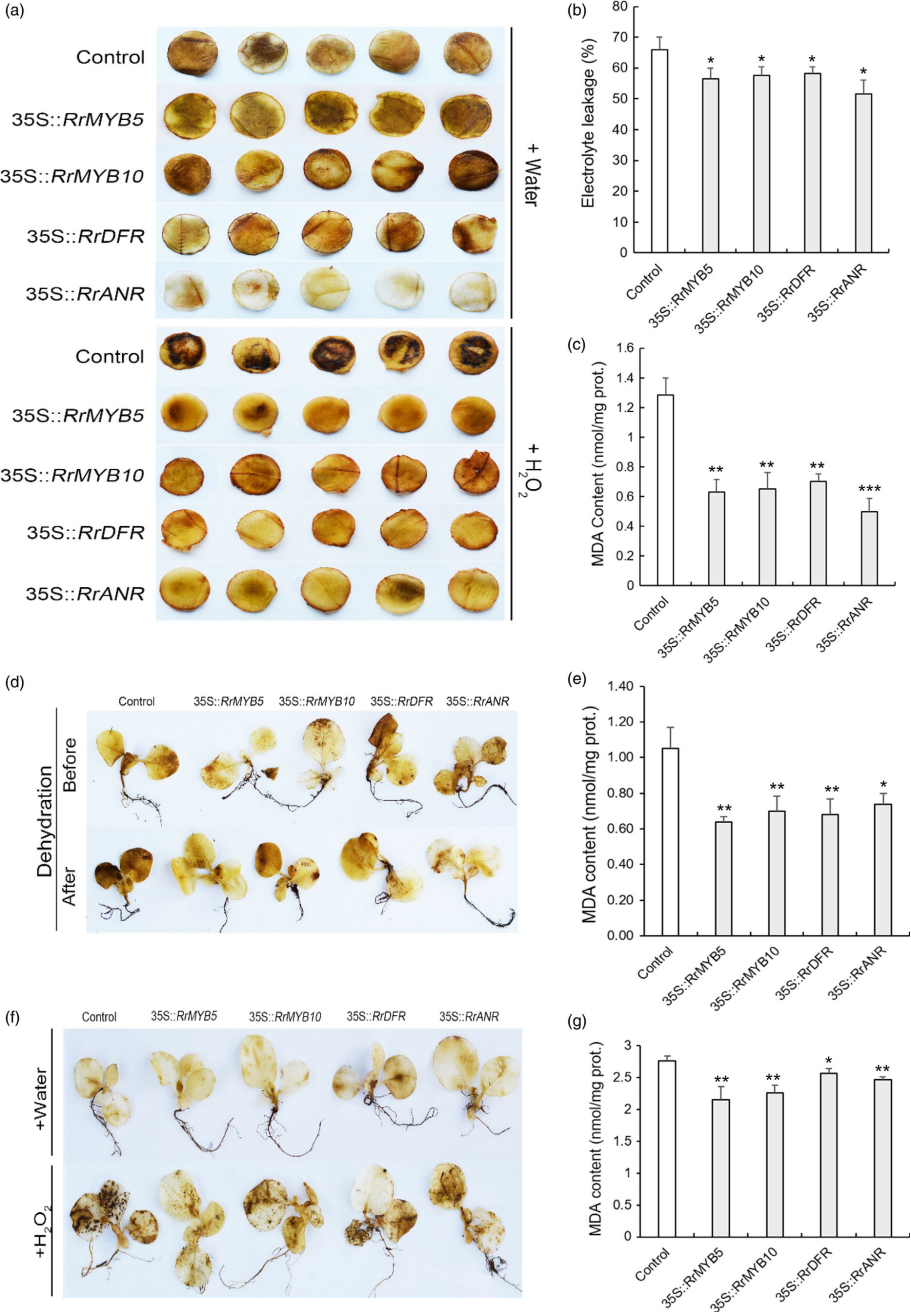
O^2−^ and H_2_O_2_ levels in *RrMYB5‐*,* RrMYB10‐, RrANR‐* and *RrDFR*‐transgenic tobacco under stress conditions. (a) Representative photographs of leaf discs of various 6‐week‐old transgenic tobacco showing staining with DAB after H_2_O_2_ treatment. (b and c) Electrolyte leakage (b) and MDA (c) levels in control and various transgenic tobaccos after H_2_O_2_ treatment. (d) Representative photographs of various 4‐week‐old transgenic tobacco showing staining with DAB under dehydration. (e) MDA levels in control and various transgenic tobaccos after dehydration. (f) Representative photographs of various 4‐week‐old transgenic tobacco showing staining with DAB after H_2_O_2_ treatment. (g) MDA levels in control and various transgenic tobaccos after H_2_O_2_ treatment. Data represent mean ± SE of three biological replicates (*n* = 3). The statistical significance was determined using Student's *t* test. (* *P* < 0.05; ** *P* < 0.01; *** *P* < 0.001).

The PA determination showed that accumulation of PAs was more significant in transgenic tobacco leaves than in controls (Figure S14). For the 6‐week‐old transgenic tobacco, DAB staining revealed no differences in ROS accumulation between controls and transgenic tobacco in the water treatment, except for 35S::*RrANR* with light staining. After 36‐h H_2_O_2_ treatment, stronger DAB staining was found in control plants than in transgenic tobacco (Figure 8a). The EL value was significantly higher in control plants (65.93%) than in the transgenic tobaccos (35S::*RrMYB5,* 56.59%; 35S::*RrMYB10*, 57.49%; 35S::*RrDFR*, 58.24%; 35S::*RrANR*, 51.67%; Figure 8b). The MDA content was dramatically reduced in the transgenic tobacco (Figure 8c). For the 4‐week‐old tobacco, the DAB staining showed no difference between control and transgenic tobacco prior to dehydration. However, the control seedlings became more intensely stained after 2‐h dehydration treatment (Figure 8d). The MDA content was dramatically reduced in the transgenic tobacco after dehydration treatment (Figure 8e). In water treatment, DAB staining showed no difference between control and transgenic tobacco seedlings, whereas in H_2_O_2_ treatment, stronger DAB staining was found in control seedlings (Figure 8f). After H_2_O_2_ treatment, MDA content was also significantly reduced in the transgenic lines (Figure 8g). In addition, for 3‐ or 4‐ week‐old seedlings, DAB and NBT staining also revealed that more ROS accumulation was found in control plants than in *RrMYB5‐* and *RrMYB10*‐transgenic tobacco seedlings with or without H_2_O_2_ treatment (Figure S15).

## Discussion

### Connections between PA accumulation, RrMYB5 and RrMYB10, and wounding and oxidation responses in *R. rugosa*

MYB TFs play pivotal roles in the response to external stimuli. MYB TFs, such as *MYB3, MYB4, MYB5, MYB102* and *MYB123,* can be induced by wounding in *Arabidopsis* (Cheong *et al*., 2002). The biosynthesis of PA has been reported to be induced by pathogen or wounding signals through the regulation of MYB TFs (Akagi *et al*., 2010; Mellway *et al*., 2009). Persimmon *DkMYB2* was induced by wounding, and it regulated PA biosynthesis (Akagi *et al*., 2010). Poplar *PtMYB115* and *PtMYB134* also responded to wounding and activated the expression of *ANR* and *LAR* to promote PA synthesis (James *et al*., 2017). The structural genes involved in PA biosynthesis were also regulated by wounding (Bhargava *et al*., 2010). These findings were consistent with our study results that wounding caused flavonoid accumulation in rose petals and leaves. The flavonoid structural genes, including *CHS, DFR, ANR and LAR*, were also up‐regulated in the wounded tissues. Coincidently, 5 MYB TFs were also significantly up‐regulated in wounded rose petals and leaves. Further studies showed that *RrMYB10* and *RrMYB5* belonged to TT2‐ and PA1‐type TFs, respectively, and that these two genes were highly up‐regulated by MV, a strong oxidizer treatment of rose leaves. Therefore, *RrMYB10* and *RrMYB5* are putative TFs specifically induced by wounding and oxidation in *R. rugosa*.

### RrMYB5 and RrMYB10 are anthocyanin or proanthocyanidin regulators

Not surprisingly, RrMYB10 showed high similarity to *AtTT2* or its homologs in its role in PA biosynthesis regulation. *MdMYB9* and *MdMYB11* were found to be related to PA accumulation in different tissues of apple. Their expression patterns were similar to that of *MdANR*, and they were all highly expressed in flowers and leaves (Gesell *et al*., 2014). In our study, *RrMYB10* had high transcript levels in *R. rugosa* flowers and leaves, so did *MdMYB11,* which was homologous to *RrMYB10*. *AtMYB5*, a MYB TF in *Arabidopsis*, was reported to have played minor roles in PA accumulation (Schaart *et al*., 2013). Few of the homologs of *AtMYB5* have been confirmed to be involved in PA biosynthesis (Deluc *et al*., 2008; James *et al*., 2017). This study indicated that the expression pattern of *RrMYB5* was similar to that of *RrANR* encoding a key enzyme in PA biosynthesis, and that both *RrMYB5* and *RrANR* were highly expressed in stems and roots. Moreover, individual overexpression of *RrMYB5* and *RrMYB10* in *R. rugosa* enhanced the accumulation of PA and promoted the expression of flavonoid‐related genes in embryos. In addition, more anthocyanins and PAs were accumulated in *R. rugosa* transgenic shoots harbouring *RrMYB5* and *RrMYB10,* respectively. Interestingly, in *RrMYB10‐*transgenic tobacco, total anthocyanin and PA content increased, while in *RrMYB5*‐transgenic tobacco, total anthocyanin decreased and PA content significantly increased. Thus, the varied anthocyanin contents led to different flower colours in transgenic tobacco. It might be attributed to the flavonoid‐related genes expression disequilibrium (such as *DFR, LAR, ANR*,* ANS* and UFGT) induced by the overexpression of *RrMYB5* and *RrMYB10*, respectively, and to the fact that these gene products competed for common substrates in order to biosynthesize anthocyanins and other flavonoid compounds. Further studies are also needed to confirm whether different flower colours result from the heterologous expression of genes or specificity of different organs and tissues in the *RrMYB5‐* and *RrMYB10*‐transgenic tobacco. The results of our study are also consistent with the previous study findings that the overexpression of *VvMYBPA1* (a homologous gene of *RrMYB5*) in tobacco plants led to light pink or white colours (Passeri *et al*., 2017). In a word, the results indicate that *RrMYB5* can promote the accumulation of PAs more effectively than other reported PA1‐type MYBs. Therefore, *RrMYB5* and *RrMYB10* could be determined as proanthocyanidin regulators.

### RrMYB5 and RrMYB10 physically interact and promote mutual expression and solely or synergistically activate structural gene expression in flavonoid biosynthesis pathways

The differential functions of the TT2‐ and PA1‐type MYB TFs have previously been reported. In poplar, PA1‐type *PtMYB115* and TT2‐type *PtMYB134* were both involved in the PA biosynthesis in leaves, and both activated themselves and each other's promoters (James *et al*., 2017). In *Medicago truncatula*, PA1‐type MtMYB5 and TT2‐type MtMYB14 formed a quaternary complex with MtTT8 and MtWD40‐1. This complex more strongly enhanced the activation of the promoters of *ANR* and LAR than individual gene (Liu *et al*., 2014). Many flavonoid‐related R2R3‐MYBs including RrMYB5 mentioned above usually exhibited strong auto‐activation activities, whereas TT2‐type RrMYB10 showed no auto‐activation activities in our study. The lack of auto‐activation activities was also observed in TT2‐type MYB LjTT2s of *Lotus japonicas* (Yoshida *et al*., 2008), SsMYB3 of *Coleus* and AtTT2 of *Arabidopsis* (Zhu *et al*., 2015). In our study, *RrMYB5* and *RrMYB10* could individually activate the expression of each other and the expression of *RrDFR*,* RrLAR* and *RrANR* (Figure 3). The individual overexpression of *RrMYB5* and *RrMYB10* in *R. rugosa* enhanced the accumulation of PA and increased the expression of flavonoid‐related genes (Figure 4). In addition, we also found that RrMYB5 could interact with RrMYB10. When *RrMYB5* and *RrMYB10* were both present, their ability to activate the downstream structural genes was reduced compared to the individual ability of these MYBs (Figure 4). The experiment with transiently transformed petals of rose indicated that the coexistence of *RrMYB5* and *RrMYB10* decreased the expression of flavonoid‐related genes. A possible reason for these findings lies in that the interaction of *RrMYB5* and *RrMYB10* affected the MYB‐bHLH‐WD40 complex formation, which in turn influenced the complex's binding to the promoters of downstream genes. Taken together, it could be concluded that *RrMYB5* and *RrMYB10* could individually promote the flavonoid accumulation in *R. rugosa* by activating the expression of flavonoid‐related genes. In addition, RrMYB10 and RrMYB5 interacted with bHLH protein EGL3, respectively, and the obtained complex also activated the promoters of *DFR, ANR* and *LAR*. Therefore, RrMYB5 and RrMYB10 also synergistically activate the promoters of structural genes in flavonoid biosynthesis pathways.

### Transcriptome of plants harbouring 35S::RrMYB10, 35S::RrDFR and 35S::RrANR

Many MYB TFs regulating flavonoid biosynthesis could be induced by wounding (Akagi *et al*., 2010; James *et al*., 2017); however, the biological significance of the phenomena was not explored. In our transgenic test, both *RrMYB10* and *RrMYB5* were induced by wounding and participated in the biosynthesis of PAs. The flavonoid‐related genes, such as *NtCHS*,* NtCHI, NtDFR* and *NtANS*, were significantly up‐regulated in the *RrMYB10‐*transgenic tobacco. We further proved that RrMYB10 protein was involved in regulation of the synthesis of flavonoids. Local damages caused by wounding, insect attack or pathogen infections cause oxidative stress in plants. Reactive oxygen species (ROS) usually acting as signalling molecules were involved in the wound‐induced production of metabolites (Choudhury *et al*., 2017). The antioxidant enzymes were involved in scavenging ROS as the first line, which include catalase (CAT), superoxide dismutase (SOD) isozymes, peroxidase (POD), ascorbate peroxidase (APX) and so on. Therefore, the expression of those genes encoding antioxidant enzymes has been found to be strongly associated with oxidative tolerance in plants (Jiang and Zhang, 2001). In our study, 21 antioxidant‐related genes, especially *NtPODs*, were significantly up‐regulated in the *RrMYB10‐*transgenic tobacco. Therefore, the above results revealed that *RrMYB10* acting as a regulatory factor activated the antioxidant system in plants. ABA signal transduction pathways were tightly associated with the responses to wounding in plants. PP2Cs (Protein phosphatase type 2C) played negative roles in ABA signalling (Schweighofer *et al*., 2004). Overexpression of *AtMYB44* enhanced drought resistance in *Arabidopsis* by repressing *PP2C* genes (Jung *et al*., 2008). Our transcriptome data showed that 14 *PP2Cs* genes were significantly down‐regulated in *RrMYB10*‐transgenic plants. Therefore, the enhanced stress resistance of *RrMYB10*‐transgenic plants might partly result from the down‐regulated *PP2Cs*.

We previously reported that the overexpression of *RrANR* enhanced tobacco tolerance to abiotic stress through the increased ability to scavenge ROS. In the *RrANR*‐transgenic tobacco, 16 antioxidant‐related genes were significantly up‐regulated (Luo *et al*., 2016). Taken together, the accumulation of flavonoids in *RrMYB10*‐transgenic tobacco leads to the enhancement of oxidative stress tolerance by increasing ROS scavenging activities. On the other hand, the consistency of expressive tendency of the genes from *RrMYB10‐* and *RrANR‐*transgenic tobacco further confirms that *RrMYB10* is the *RrANR* upstream regulator.

### Overexpression of RrMYB5 and RrMYB10 resulted in high flavonoid accumulation and enhanced tolerance

Overexpression of TT2‐ or PA1‐type MYB genes promoted the expression of the genes involved in PA accumulation (Tian *et al*., 2017; Wang *et al*., 2018). In our study, overexpression of the PA1‐type *RrMYB5* and TT2‐type *RrMYB10*, whether in *R. rugosa* or tobacco, all resulted in higher PA accumulation in both transgenic plants. The accumulation of flavonoids enhanced plant tolerance to abiotic stresses (Lotkowska *et al*., 2015), which was consistent with our study results that the *RrMYB5‐* and *RrMYB10‐*transgenic roses exhibited a lower ROS level and higher level of SOD and CAT activities than wild type, and these transgenic roses displayed the enhanced tolerance to oxidative stress. Our previous studies showed that overexpression of *RrDFR* and *RrANR* in tobacco resulted in more PA accumulation, the enhanced oxidative stress tolerance (Luo *et al*., 2016). *RrMYB5‐, RrMYB10‐, RrDFR‐* and *RrANR*‐transgenic tobacco all displayed the enhanced tolerance to oxidation and dehydration. These transgenic tobacco plants exhibited lower ROS levels than the control tobacco plants under either dehydration or H_2_O_2_ treatments. These results suggested that *RrMYB5* and *RrMYB10* overexpression enhanced the tolerance against oxidative stress by promoting *RrDFR* and *RrANR* expression and accumulating more PAs in transgenic plants.

### A model of flavonoid‐mediated feedback loop responding to wounding and oxidative stress in *Rosa rugosa*

**Figure 9:**
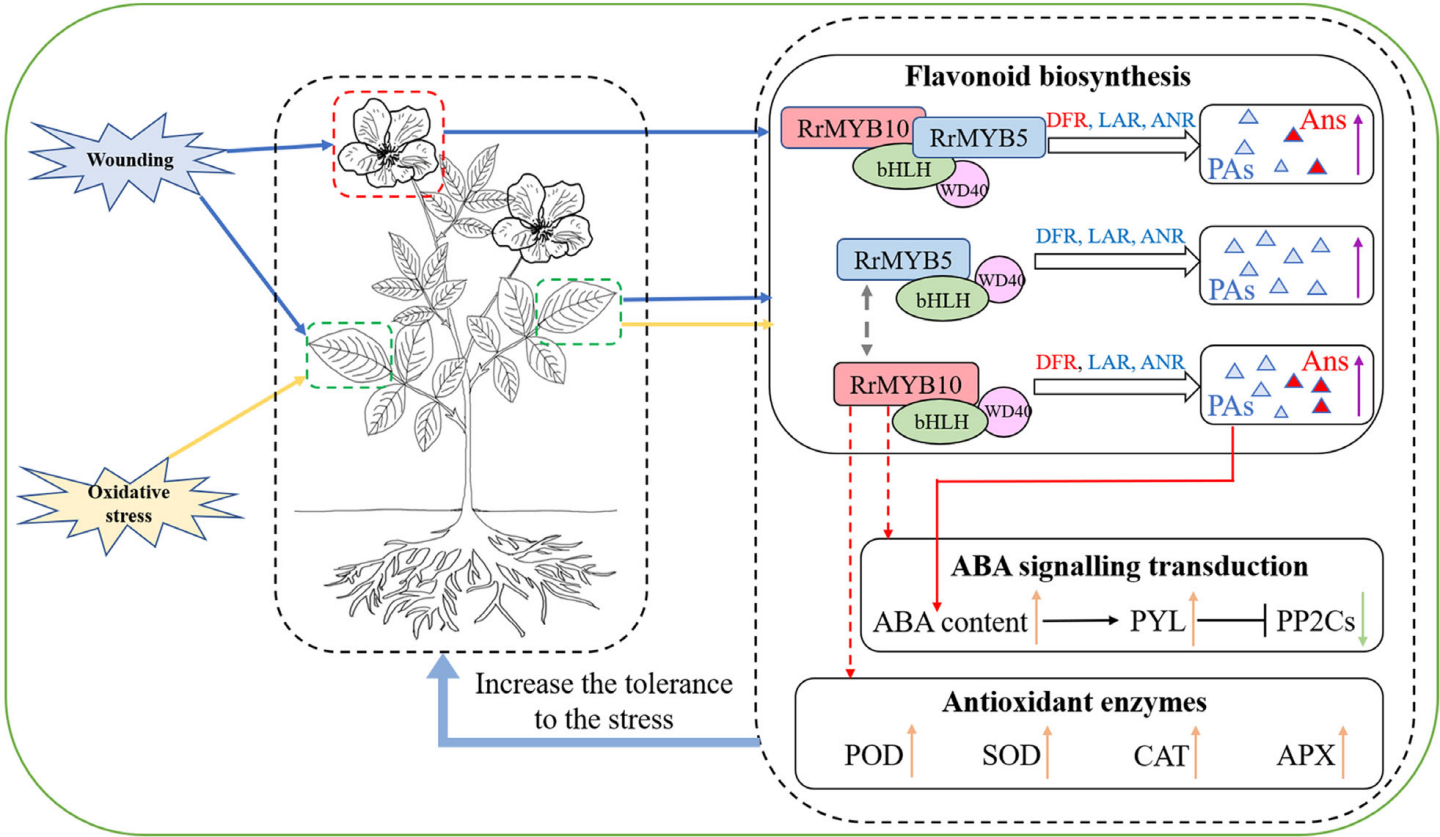
Model of transcriptional regulatory network controlling PA biosynthesis in response to wounding and oxidative damage in *Rosa rugosa*. The blue and yellow arrows indicate that wounding and oxidation induce the expression of *RrMYB5* and *RrMYB10* in flowers and leaves of rose. The grey dotted arrow represents the positive activation of the *RrMYBs’* expression. The purple arrows represent the flavonoids accumulated in flowers and leaves of rose. The orange arrows represent gene up‐regulation in *RrMYB10*‐transgenic tobacco, while the green arrows represent down‐regulation. The red dotted arrows indicate that ABA signalling transduction genes and antioxidant enzymes are influenced by *RrMYB10* according to the transcriptome profiles. The red arrows indicate that PAs enhance the accumulation of ABA [as described in our previous study (Luo *et al*., 2016)]. The blue triangles represent the PAs, and red triangles represent the anthocyanins.

*R. rugosa*, as a commercially important gardening plant, often suffers from various abiotic and biotic stresses. Our study showed that two wounding‐ or oxidation‐induced R2R3 MYB TFs, *RrMYB5* and *RrMYB10*, regulated the flavonoid biosynthesis genes and mediated the wounding‐ and oxidation‐induced PA accumulation in rose plants, that RrMYB5 and RrMYB10 physically interacted and mutually regulated the expression of each other, and that these two genes solely and synergistically activated the promoters of key structural genes involved in flavonoid biosynthesis. The overexpression of *RrMYB5* and *RrMYB10*, or their targeted genes, *RrDFR* and *RrANR,* increased the production of anthocyanins or proanthocyanidins in transgenic *R. rugosa* or tobacco, and enhanced the tolerance to wounding and oxidative stresses by increasing ROS scavenging and modulating ABA signalling (Figure 9). Therefore, a model of feedback loop mediated by flavonoid in response to wounding and oxidation was proposed. This model explains that repeated cutting rose flowers and gardening pruning may enhance the overall tolerance against various abiotic and biotic stresses in *R. rugosa*.

## Experimental procedures

### Plant materials and growth conditions

Plants of the *Rosa rugosa* cultivars ‘Zizhi’ and ‘Bao White’ grown at Huazhong Agricultural University were sampled. Total RNA was extracted using the adapted CTAB method as previously described (Luo *et al*., 2016). Transformed tobacco plants were grown in a greenhouse with a 16‐/8‐h light/dark photoperiod and with a 25/22 °C day/night temperature regime.

### Phylogenetic analysis and isolation of RrMYB5 and RrMYB10

Transcriptome data of *R. rogusa* were *de novo* assembled by TRINITY (Grabherr *et al*., 2011). Protein sequences were predicted using TransDecoder (http://transdecoder.github.io). A total of 36 *Arabidopsis* MYB genes involved in stress response were selected from the TAIR (Arabidopsis Information Resource; Table S1). The Arabidopsis MYB proteins were used to perform homologs analysis with respect to the *Fragaria vesca* genome database (Phytozome database, *Fragaria vesca* v1.1) and *R. rogusa* transcriptome data sets. Protein alignment and phylogenetic analyses were performed as described previously by us (Wang *et al*., 2017). Genes or promoter fragments were amplified with gene‐specific primers (Table S2). Amplified products were cloned into a modified vector pCAMBIA2300s containing the 35S promoter and the NOS terminator.

### Real‐time PCR analysis

qRT‐PCRs were performed using an Applied Biosystems (CA) 7500 real‐time PCR machine. The reaction mixture and program conditions were set as described previously by us (Wang *et al*., 2017). The housekeeping gene (*RrGADPH* or *NtEF‐1‐*α) was used as an internal standard. Primers for RT‐PCR and qRT‐PCR are presented in Table S3.

### Subcellular localization analysis

The ORF of *RrMYB5* and *RrMYB10* (without stop codon) was amplified with the primers (Table S2) and cloned into *pLGFP1301* to create 35S::*RrMYB5:GFP* and 35S::*RrMYB10:GFP* constructs. The constructs were used for transient assay by *Agrobacterium‐mediated* transfection of 5‐ to 6‐week‐old *N*. *benthamiana* leaves (Li *et al*., 2015). After 2‐day culture, the samples were observed with a fluorescence microscope (LEICA, DM2500).

### Wounding, methyl viologen (MV) and light treatments

For wounding treatment, the flower buds and leaves were first incubated in distilled water at room temperature. After 2 days, leaves and petals were cut with scissors. Wounded leaves and middle‐round petals were collected 24 h after wounding and were frozen in liquid nitrogen and then stored at −80 °C prior to analysis.

For methyl viologen (MV) treatment, shoots (Figure S16) were first incubated in distilled water for 48 h at 25 °C with a photoperiod of 16‐/8‐h light/dark and then treated with MV. The leaves were collected at 0, 6, 12, 24, 48 and 72 h after culture with 0 or 100 μm MV, immediately frozen in liquid nitrogen and then stored at −80 °C until used.

For light treatment, tissue‐cultured seedlings of *R. rugosa* (Figure S15) were grown in complete darkness for 3 days. Subsequently, the treatment groups were grown under fluorescent lamps in a 16‐h light/8‐h dark photoperiod at 600 μmol/m^2^/s at 22 °C for 3 days. The controls were grown in darkness. Whole seedlings were sampled to analyse gene expression and measure the contents of anthocyanin and PAs.

### Yeast two‐hybrid assays and split luciferase complementation assays

For yeast two‐hybrid assays, the CDSs of *RrMYB5* and *RrMYB10* were recombined into *pGADT7*, and those of *AtEGL3* and *RrMYB10* were recombined into *pGBKT7*. The full‐length ORF and two truncated (*RrMYB5*_*1‐242*_, *RrMYB5*_*1‐216*_, shown in Figure S5A) ORF fragments of *RrMYB5* were amplified with primers (Table S2) and recombined into *pGBKT7*. The yeast two‐hybrid assays were performed as previously described by us (Wang *et al*., 2017).

For split luciferase complementation assays, the CDS of *RrMYB10* (without stop codon) was amplified with primers (Table S2) and recombined into *pJW771‐NLuc*, and that of *RrMYB5* (without stop codon) was recombined into *pJW772‐CLuc*; then, the constructs were introduced into the *Agrobacterium tumefaciens* GV3101 strain. Infiltration of 5‐ to 6‐week‐old *N. benthamiana* leaves and detection of luminescence signals followed the protocol described in Zhang *et al*. (2018). For the infiltration of tobacco leaves, one‐half of the leaf infected with RrMYB5‐CLuc + RrMYB10‐NLuc was used as the experimental group, and the other half injected with RrMYB5‐CLuc + NLuc, or RrMYB10‐NLuc + CLuc, or NLuc + CLuc was used as the control group. After 48‐h infiltration, the leaves were sprayed with 1 mm luciferin substrate (Gold Bio) and luminescence signals were acquired by a CCD imaging apparatus (Lumazone Pylon2048B).

### Dual‐luciferase reporter assay

For the dual‐luciferase reporter assay, the CDSs of *RrMYB5, RrMYB10* and *AtEGL3* were amplified with primers (Table S2) and combined into the *pGreenII 62‐SK* effector vector under the control of CaMV 35S. The *RrDFR*,* RrLAR*,* RrANR*,* RrMYB5* and *RrMYB10* promoter fragments were ligated into the reporter vector *pGreenII 0800‐LUC*. *Arabidopsis* protoplast isolation and polyethylene glycol‐mediated co‐transformation of the effector and reporter constructs were performed as previously described (Li *et al*., 2015). The transformed protoplasts were incubated for 16 h at 22 °C before activity assay. The Dual‐Luciferase Reporter Assay System (Promega, E1910) was used according to manufacturer's instruction with an Infinite200 Pro microplate reader (Tecan). The promoter activity was expressed as a ratio of LUC to REN.

### Transformation of rose and tobacco

For transformation, the CDSs of *RrMYB5* and *RrMYB10* were recombined into *pCAMBIA 2300s* and then transformed into *A. tumefaciens* EHA105. The *Rosa rugosa* and tobacco transformation was performed as previously described by us (Ning *et al*., 2012; Xing *et al*., 2014). After 2‐month culture, somatic embryos of *R. rugosa* were sampled to analyse gene expression and measure the content of PAs. After 6–8‐month culture, the germinated shoots were used for oxidative stress tolerance assays.

### Transiently transformed petals of *R. hybrida*

The CDSs of *RrMYB10, RrMYB5* and *AtEGL3* were recombined into *pCAMBIA 2300s* and then introduced into the *Agrobacterium tumefaciens* GV3101 strain. The empty *pCAMBIA 2300s* vector was used as the negative control. The infiltration of *R. hybrida* petals was according to the protocol described by Yasmin and Debener (2010).

### Determination of anthocyanin and proanthocyanidin levels

The determination of anthocyanin content was conducted as previously described (Luo *et al*., 2015). The level of anthocyanins was determined by the formula: *Q*_Anthocyanins_ = (A530–0.25 × *A*_657_) × M^−1^.

Soluble PA determination was performed as previously described (Luo *et al*., 2016). The obtained products were quantified by reacting with DMACA (p‐dimethylamino‐cinnamaldehyde). The level of PAs was determined according to Liu *et al*. (2013). The total PA levels were calculated as procyanidin equivalents using procyanidin B2 (Sigma‐Aldrich, MO) as standards.

### Transcriptomic sequencing analysis

Illumina sequencing and *de novo* assembly in *R. rogusa* (Tables S5 and S6) were conducted as previously described by us (Ning *et al*., 2017). Genome‐guided transcript assembly was performed to obtain a transcript data set for RNA‐seq analysis in tobacco (Table S7). Hisat2 was used to align reads against the genome (Sierro *et al*., 2014) with the default parameters (Kim *et al*., 2015). Cufflinks was used to individually generate GTF files, and cuffmerge was used to merge total GTF files. PASA was used to generate the transcript data sets. Cufflinks was used to quantify the expression level of each transcript. The differentially expressed genes (DEG) were determined based on a false discovery rate (FDR) threshold of <0.05, an absolute log2‐fold change value of >1.0 and a *P*‐value < 0.05. As indicated in the figure legends, we conducted log2 transformations as recommended by Pertea *et al*. (2016). Transcript annotation was performed against the NCBI nonredundant (nr) database, the Swiss‐Prot protein database, and the Kyoto Encyclopedia of Genes and Genomes (KEGG) database (Moriya *et al*., 2007) using BLASTx with an *E*‐value ≤10^−5^. The WEGO online tool (Ye *et al*., 2006) was used to classify GO functions and calculate the distribution of gene functions at the systematic level.

### Analysis of dehydration and oxidative stresses

For the oxidative stress treatment of *R. rugosa*, the 9–12 tissue‐cultured shoots about 2–3 cm (wild type and transgenic) were submerged in 2% H_2_O_2_ for 30 h. Then, shoots were mixed, sampled, and subsequently used for measuring MDA content, and SOD and CAT activities, and used for histochemical staining (DAB). The MDA content, and SOD and CAT activities were measured using specific detection kit following the manufacturer's instructions (A003‐1 for MDA, A001‐1 for SOD and A007‐1 for CAT; Nanjing Jiancheng Bioengineering Institute, China).

Four‐week‐old tobacco seedlings were dehydrated for 2 h on filter paper as previously described (Luo *et al*., 2016). The treated seedlings were sampled to measure MDA content, and seedlings were subjected to histochemical staining with DAB.

For the oxidative stress treatment of tobacco, leaf discs were sampled and the experiments were undertaken as previously described (Luo *et al*., 2016). After treatments, MDA content and electrolyte leakage (EL) were determined. Histochemical staining was conducted with DAB. In addition, 3–4‐week‐old tobacco seedlings were incubated in 2% H_2_O_2_, or in water, for 48 h. Finally, the MDA content and histochemical staining for H_2_O_2_ (DAB) or O_2_^−^ (NBT) were determined.

## Conflict of interest

The authors declare no conflict of interest.

## Supporting information


**Figure S1** Phylogenetic analysis of selected MYB transcript factors.
**Figure S2** Sequence alignment of RrMYB5, RrMYB10 and R2R3‐MYB transcription factors from various plant species.
**Figure S3** Characterization of *RrMYB5* and *RrMYB10* promoters and the effect of light on expression.
**Figure S4** Subcellular localization of RrMYB5‐GFP and RrMYB10‐GFP in leaves of *Nicotiana benthamiana*.
**Figure S5** Protein interaction analysis between RrMYB5 and RrMYB10 by yeast two‐hybrid assays and split luciferase complementation assays.
**Figure S6** Gene activation analysis of *RrMYB5*,* RrMYB10, AtEGL3* and *AtTTG1* using a dual‐luciferase assay in *Arabidopsis* protoplast.
**Figure S7** Phenotype of varied transgenic tobaccos used to perform RNA‐Seq analysis.
**Figure S8** Histogram of gene ontology (GO) categorization for the differentially expressed genes between the wild type and *RrMYB10* transgenic tobacco lines.
**Figure S9** Histogram of gene ontology (GO) categorization for the differentially expressed genes between the wild type and *RrANR* transgenic tobacco lines.
**Figure S10** Histogram of gene ontology (GO) categorization for the differentially expressed genes between the wild type and *RrDFR* transgenic tobacco lines.
**Figure S11** Comparisons of differentially expressed genes between RrMYB10 with RrANR and RrMYB10 with RrDFR transgenic tobacco.
**Figure S12** Common differentially expressed genes associated with hormone metabolism and signaling in varied transgenic tobaccos.
**Figure S13** Common differentially expressed genes belonging to antioxidant‐related genes in varied transgenic tobaccos.
**Figure S14** Phenotype and PA content measured in *RrMYB5, RrMYB10*,* RrANR* and *RrDFR* transgenic tobacco leaves.
**Figure S15** Representative photos of various 3‐week‐old transgenic tobacco plants harboring *35S::RrMYB5* and *35S::RrMYB10* showing staining with NBT (A) and DAB (B) after H_2_O_2_ treatment.
**Figure S16** Plants tested in our experiments were propagated from one genotype by tissue culture.
**Table S1** Stress‐response related MYB genes previously reported in *Arabidopsis*.
**Table S2** Primers used to construct vectors.
**Table S3** Primers used for qRT‐PCR and semiquantitative RT‐PCR analysis.
**Table S5** Summary of sample short reads from SGS sequencing of *Rosa rugosa* after clearing.
**Table S6** Summary of transcripts assembled from *Rosa Rugosa* short‐read data using Trinity.
**Table S7** Summary of sample short reads from SGS sequencing of tobacco after clearing.

**Table S4** Amino acid sequences from strawberry, *Arabidopsis*, and *R. rugosa* MYB transcription factors and used for Phylogenetic analysis.

**Table S8** Up‐ and downregulated genes in transgenic tobacco overexpressing *RrMYB10*.

**Table S9** Up‐ and downregulated genes in transgenic tobacco overexpressing *RrDFR*

**Table S10** Up‐ and downregulated genes in transgenic tobacco overexpressing *RrANR*.

## Data Availability

RNA‐Seq data generated in this study have been uploaded to BioProject at NCBI with the accession numbers PRJNA498442 (*Rosa rugosa*) and PRJNA498348 (tobacco). All other relevant data are available in this article and its supplementary files.
