# Supplementary material for: RrMYB5‐ and RrMYB10‐regulated flavonoid biosynthesis plays a pivotal role in feedback loop responding to wounding and oxidation in Rosa rugosa
Source: Plant Biotechnol J. 2019 Apr 14;17(11):2078–95. doi: 10.1111/pbi.13123 (PMC6790370; doi:10.1111/pbi.13123)
Supplement: Supplementary file 1 — Figure S1 Phylogenetic analysis of selected MYB transcript factors. Figure S2 Sequence alignment of RrMYB5, RrMYB10 and R2R3‐MYB transcription factors from various plant species. Figure S3 Characterization of RrMYB5 and RrMYB10 promoters and the effect of light on expression. Figure S4 Subcellular localization of RrMYB5‐GFP and RrMYB10‐GFP in leaves of Nicotiana benthamiana. Figure S5 Protein interaction analysis between RrMYB5 and RrMYB10 by yeast two‐hybrid assays and split luciferase complementation assays. Figure S6 Gene activation analysis of RrMYB5, RrMYB10, AtEGL3 and AtTTG1 using a dual‐luciferase assay in Arabidopsis protoplast. Figure S7 Phenotype of varied transgenic tobaccos used to perform RNA‐Seq analysis. Figure S8 Histogram of gene ontology (GO) categorization for the differentially expressed genes between the wild type and RrMYB10 transgenic tobacco lines. Figure S9 Histogram of gene ontology (GO) categorization for the differentially expressed genes between the wild type and RrANR transgenic tobacco lines. Figure S10 Histogram of gene ontology (GO) categorization for the differentially expressed genes between the wild type and RrDFR transgenic tobacco lines. Figure S11 Comparisons of differentially expressed genes between RrMYB10 with RrANR and RrMYB10 with RrDFR transgenic tobacco. Figure S12 Common differentially expressed genes associated with hormone metabolism and signaling in varied transgenic tobaccos. Figure S13 Common differentially expressed genes belonging to antioxidant‐related genes in varied transgenic tobaccos. Figure S14 Phenotype and PA content measured in RrMYB5, RrMYB10, RrANR and RrDFR transgenic tobacco leaves. Figure S15 Representative photos of various 3‐week‐old transgenic tobacco plants harboring 35S::RrMYB5 and 35S::RrMYB10 showing staining with NBT (A) and DAB (B) after H2O2 treatment. Figure S16 Plants tested in our experiments were propagated from one genotype by tissue culture. Table S1 Stress‐response related MYB gene [file PBI-17-2078-s004.pdf]

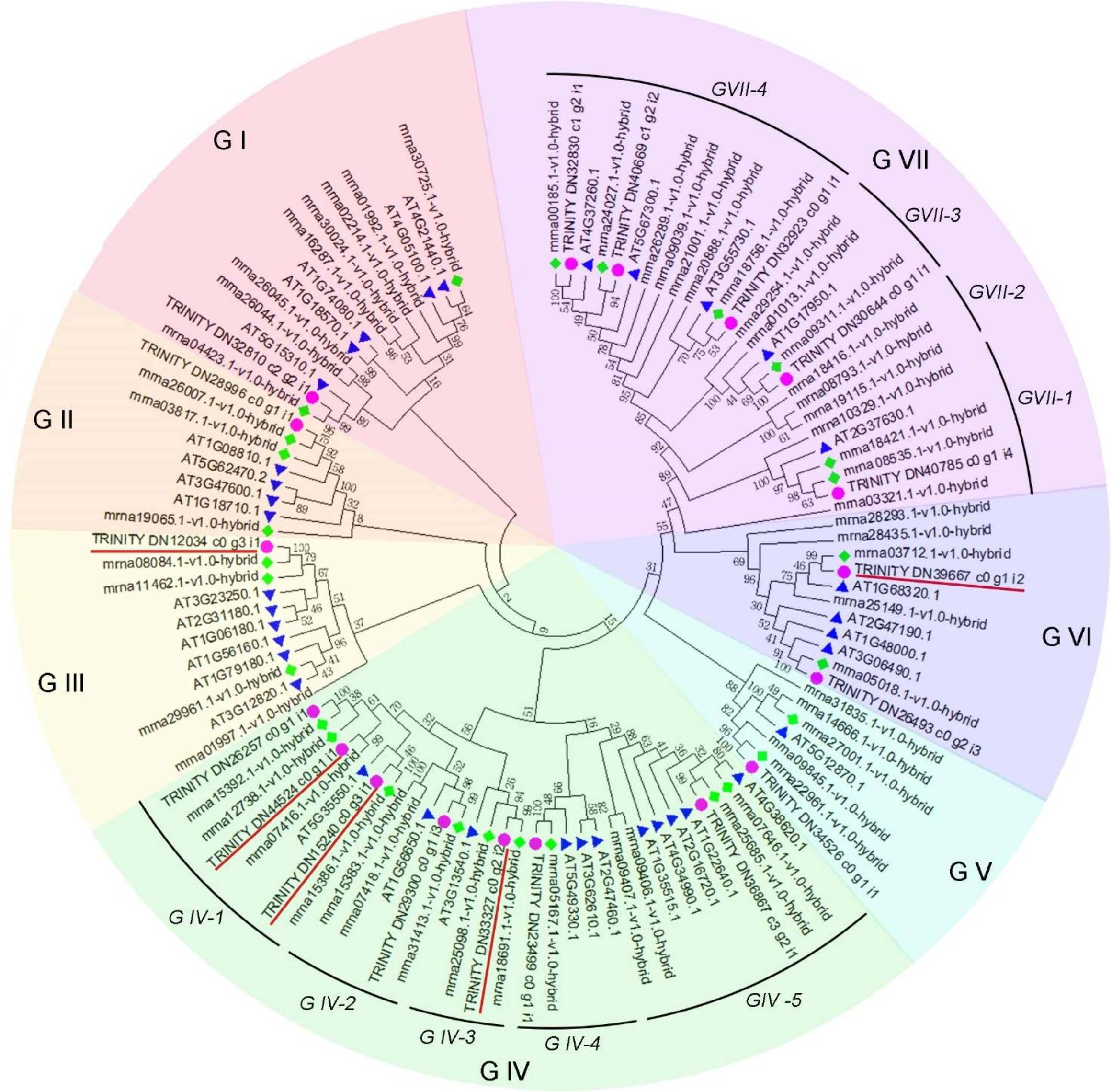

Figure S1: Phylogenetic analysis of selected MYB transcript factors. 18 *Rosa rugosa* MYB genes, 56 *Fragaria vesca* MYB genes, and 36 *Arabidopsis* MYB genes were used in the phylogenetic tree construction. The 18 rose MYB genes were obtained from the transcriptome of *R. rugosa* ‘Bao White’; 36 *Arabidopsis* MYB genes, reported to be involved in stress response, were selected from the TAIR database (*Arabidopsis Information Resource*), and 56 strawberry MYB genes were obtained from the *Phytozome database* (*Fragaria vesca* v1.1 genome). The blue triangles represent the response-related genes in *Arabidopsis thaliana*. Purple circles represent the candidate stress response MYB genes from *R. rugosa*. Green rhombuses represent the putative stress response MYB genes from *F. vesca*. Red lines represent the candidate MYB genes verified by qRT-PCR in *R. rugosa*.

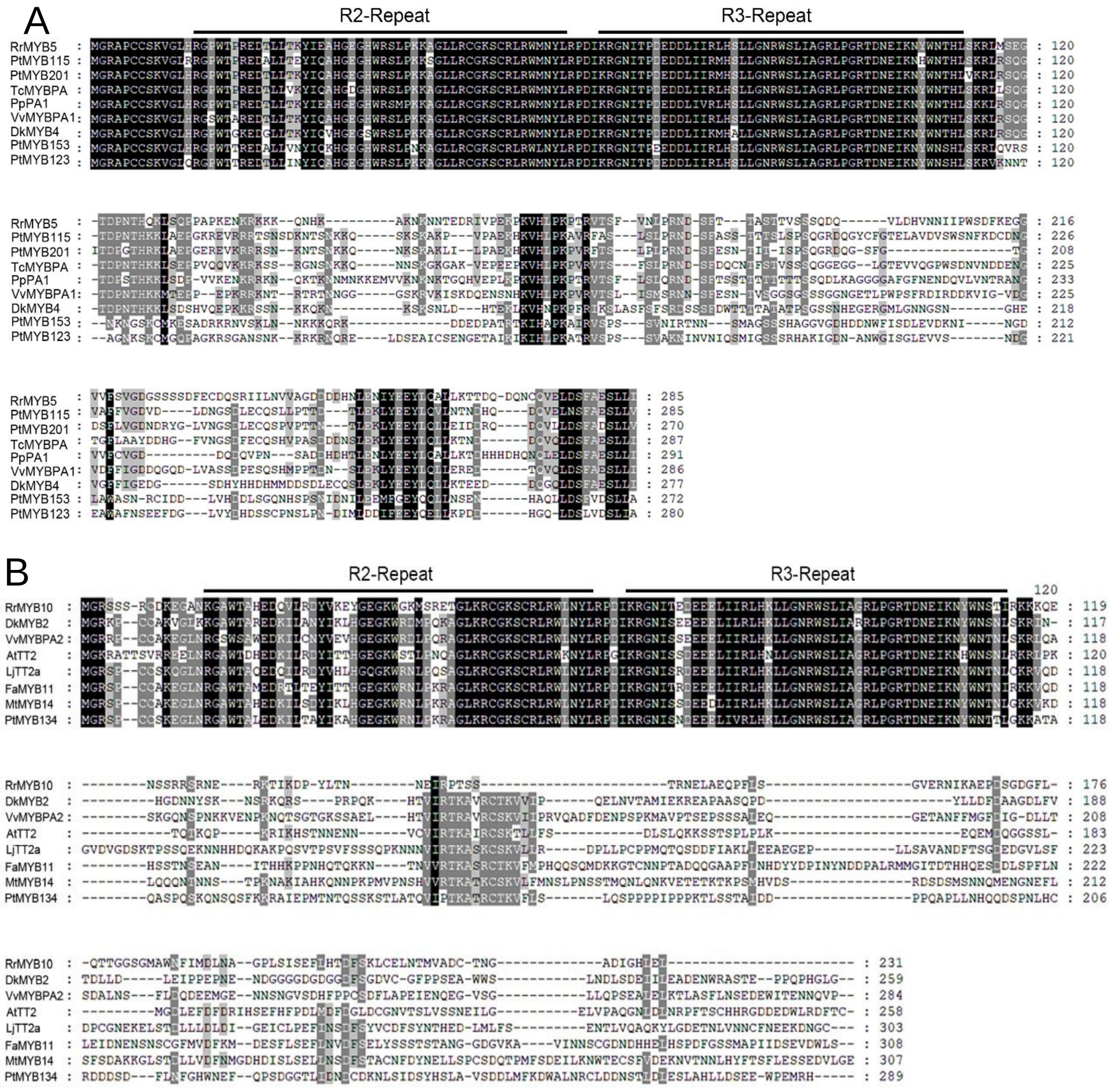

Figure S2: Sequence alignment of RrMYB5, RrMYB10 and R2R3-MYB transcription factors from various plant species. (A) Alignment of protein sequences from RrMYB5 and R2R3-MYB transcription factors from various plant species. (B) Alignment of protein sequences from RrMYB10 and R2R3-MYB transcription factors from various plant species. Black lines indicate the DNA binding domain corresponding to R2R3-MYB repeats.

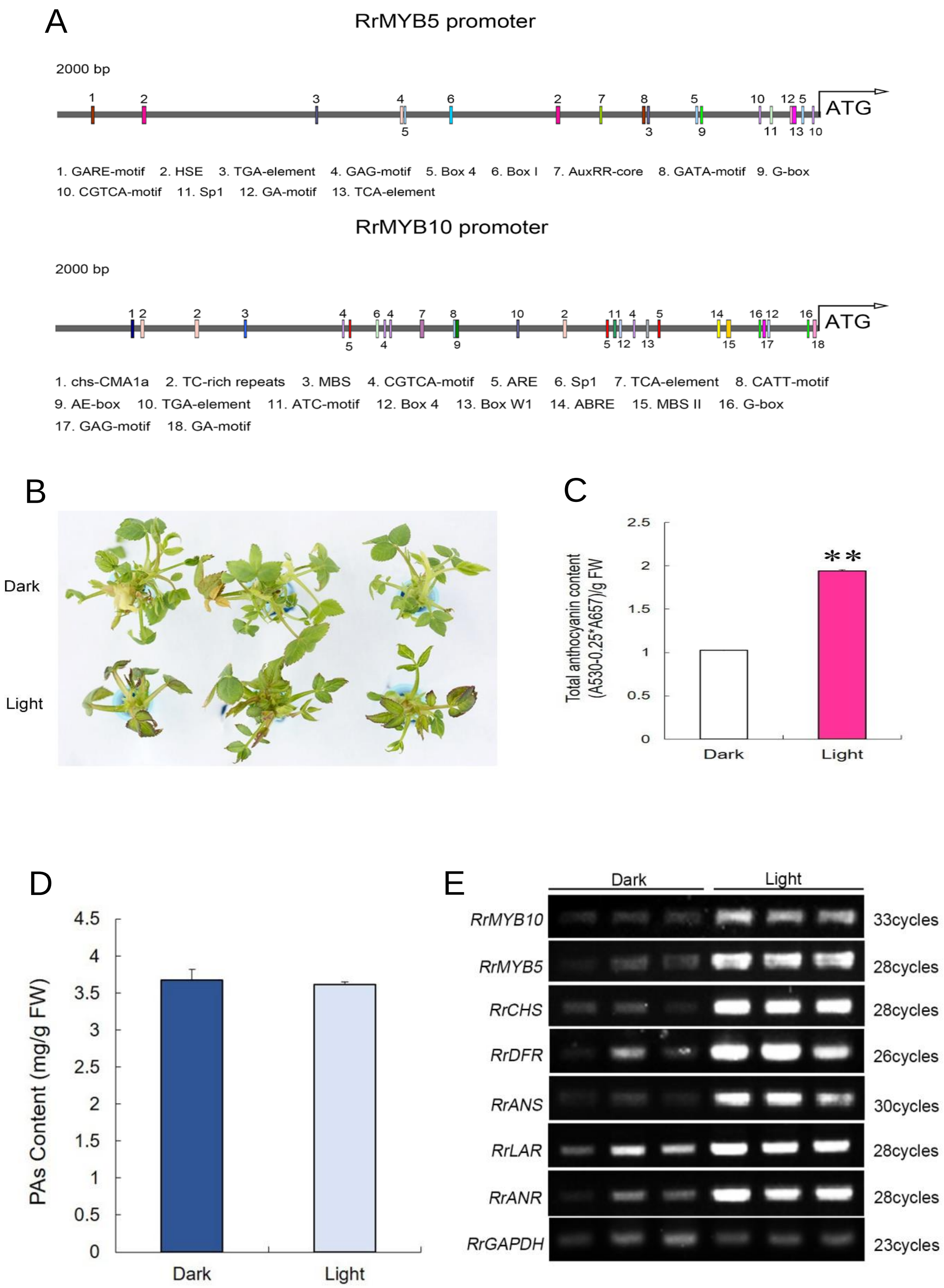
A
RrMYB5 promoter
2000 bp
ATG
1. GARE-motif 2. HSE 3. TGA-element 4. GAG-motif 5. Box 4 6. Box I 7. AuxRR-core 8. GATA-motif 9. G-box
10. CGTCA-motif 11. Sp1 12. GA-motif 13. TCA-element
RrMYB10 promoter
2000 bp
ATG
1. chs-CMA1a 2. TC-rich repeats 3. MBS 4. CGTCA-motif 5. ARE 6. Sp1 7. TCA-element 8. CATT-motif
9. AE-box 10. TGA-element 11. ATC-motif 12. Box 4 13. Box W1 14. ABRE 15. MBS II 16. G-box
17. GAG-motif 18. GA-motif
B
Dark
Light
C
Total anthocyanin content (A530-0.25*A657)/g FW
**
Dark
Light
D
PAs Content (mg/g FW)
Dark
Light
E
Dark
Light
RrMYB10 33cycles
RrMYB5 28cycles
RrCHS 28cycles
RrDFR 26cycles
RrANS 30cycles
RrLAR 28cycles
RrANR 28cycles
RrGAPDH 23cycles

Figure S3: Characterization of *RrMYB5* and *RrMYB10* promoters and the effect of light on expression. (A) Characteristics of cis-elements in *RrMYB5* and *RrMYB10* promoters by using *PlantCARE* database analysis. (B) Seedlings of *R. rugosa* after 3 days light exposure. (C-D) Anthocyanin (C) and PAs contents (D) in seedlings after 3 days light exposure. (E) Expression of *RrMYB5*, *RrMYB10* and flavonoid biosynthesis-related genes in light treated seedlings by Semiquantitative RT-PCR analysis. *RrGAPDH* was used as an internal control gene. Data represent mean ± SE of three biological replicates (n = 3). The statistical significance was determined using Student's t test. (* $P<0.05$; ** $P<0.01$).

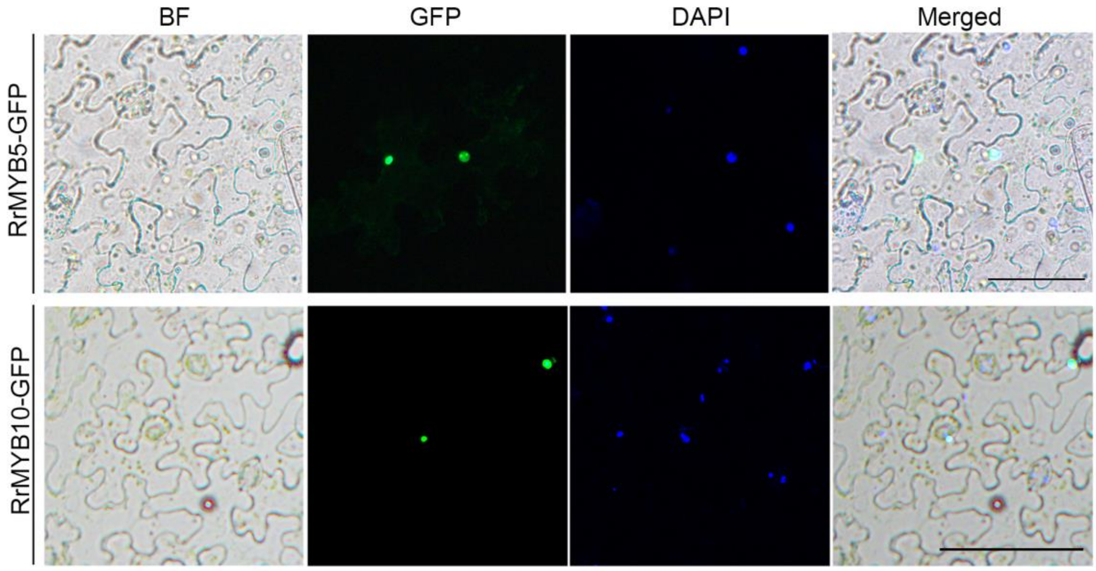

Figure S4: Subcellular localization of RrMYB5-GFP and RrMYB10-GFP in leaves of *Nicotiana benthamiana*. Both fusion proteins are localized to nuclei. BF: bright field; DAPI: nucleus stained by DAPI; Merged: merged images of bright filed, GFP fluorescence and nucleus fluorescence. Scale bar = 5 μm.

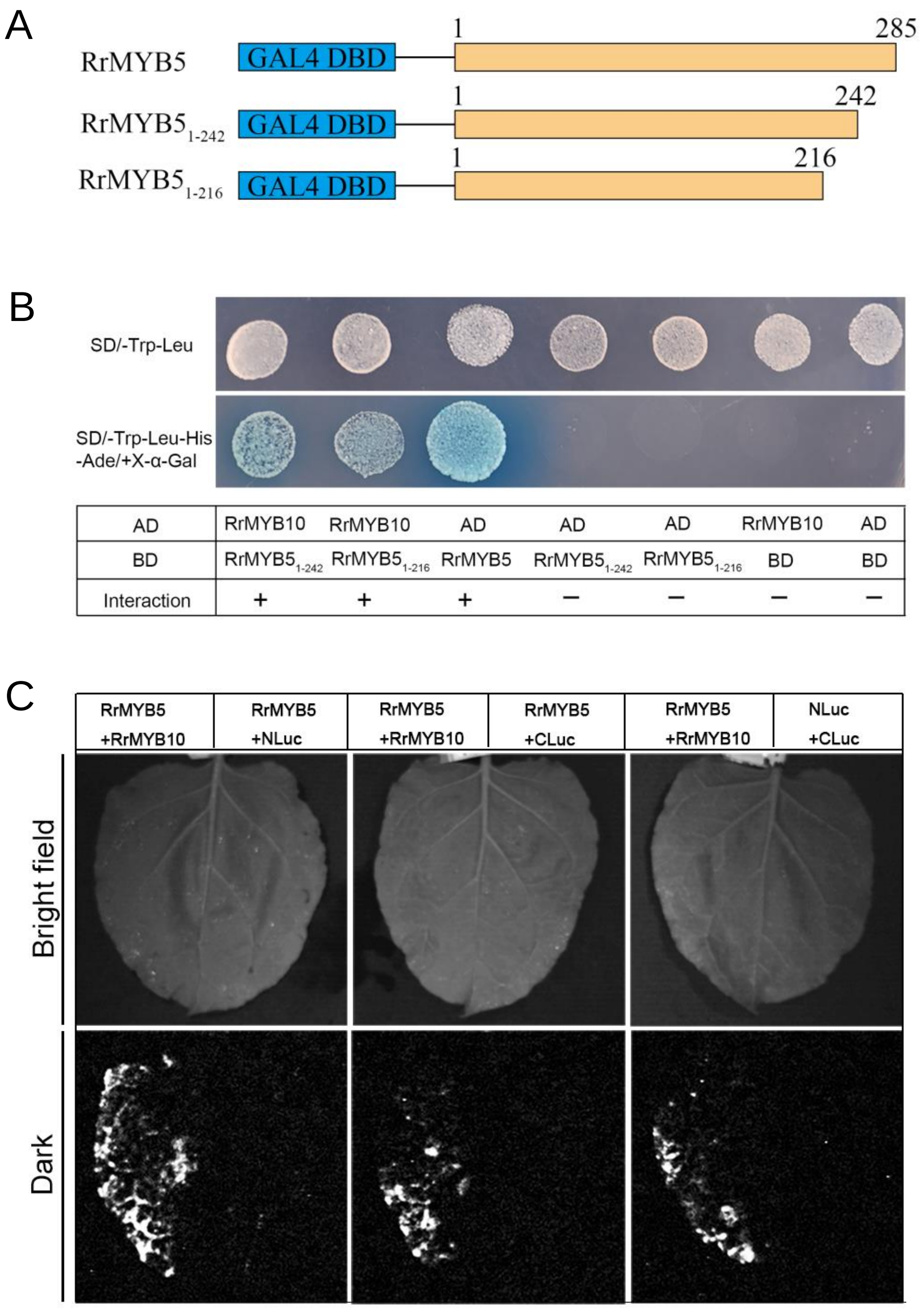

Figure S5: Protein interaction analysis between RrMYB5 and RrMYB10 by yeast two-hybrid assays and split luciferase complementation assays. (A) Schematic diagrams of constructs used for yeast two-hybrid assays. (B) The two truncated RrMYB5 interact with RrMYB10, respectively. The numbers in the RrMYB5$_{1\text{-}242}$ and RrMYB5$_{1\text{-}216}$ indicate the length of proteins after deletion of the N terminus. RrMYB5 indicates the full-length amino acid sequence. The full-length RrMYB5 protein has the self-activation in yeast two-hybrid assays. (C) RrMYB5 interacts with RrMYB10 in split luciferase complementation assays. The positive luminescence monitored by a CCD camera indicate interaction. The bright field and dark photos are merged and shown in the Figure 3B.

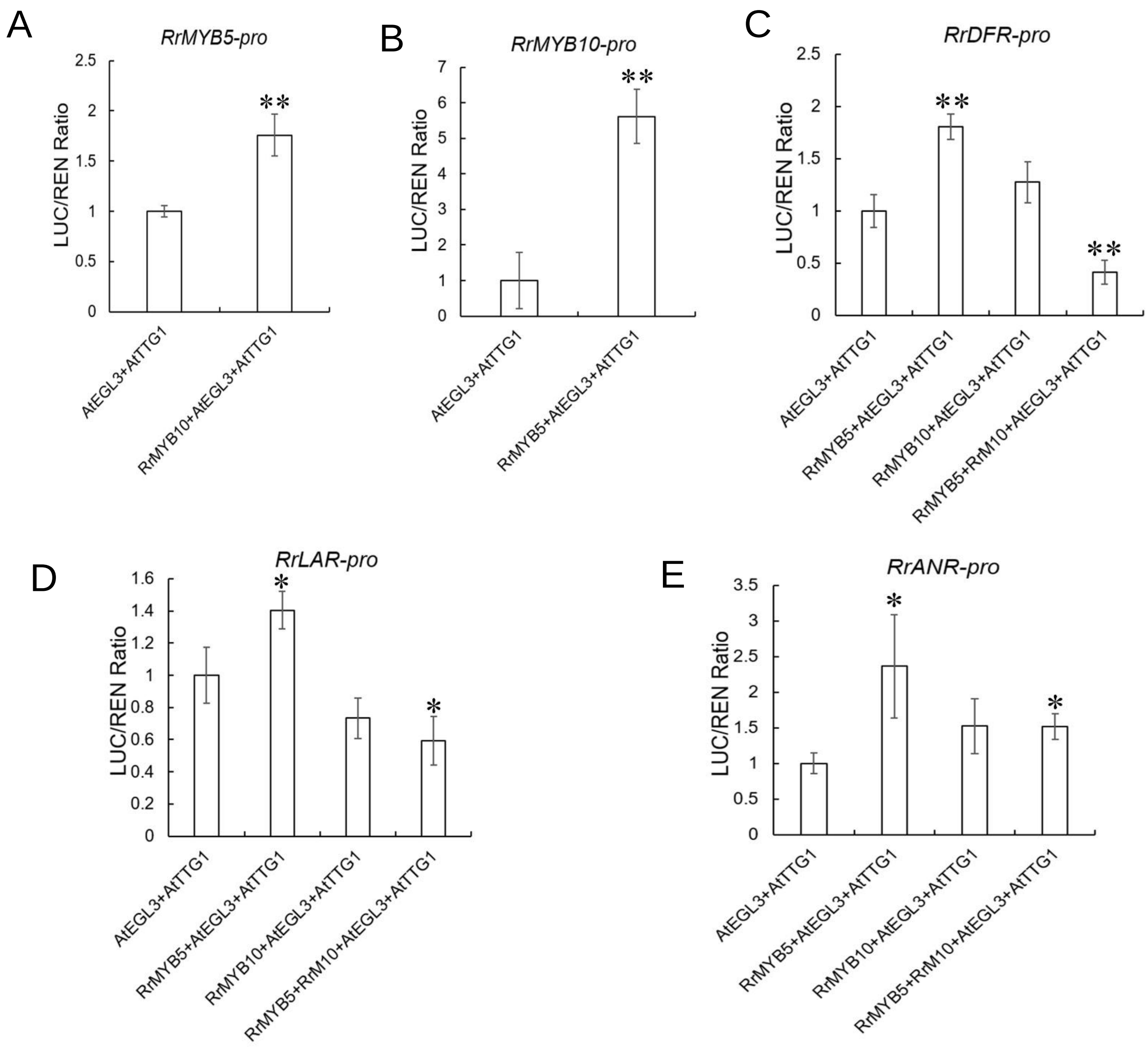

Figure S6: Gene activation analysis of *RrMYB5, RrMYB10, AtEGL3* and *AtTTG1* using a dual-luciferase assay in *Arabidopsis* protoplast. (A-B) *RrMYB10* (A) and *RrMYB5* (B) MBW complexes mutually activate the expression of each other. (C-E) *RrMYB5* and *RrMYB10* MBW complexes alone or synergistically activate the expression of *RrDFR, RrLAR* and *RrANR*. *AtEGL3* and *AtTTG1* were used as controls. The reporters and effectors were coexpressed in *Arabidopsis* protoplast, and both REN and LUC activities were measured. Relative LUC activities normalized to the REN activities are shown (LUC/REN). Data represent mean ± SE of three biological replicates (n = 3). The statistical significance was determined using Student's t test. (* $P<0.05$; ** $P<0.01$).

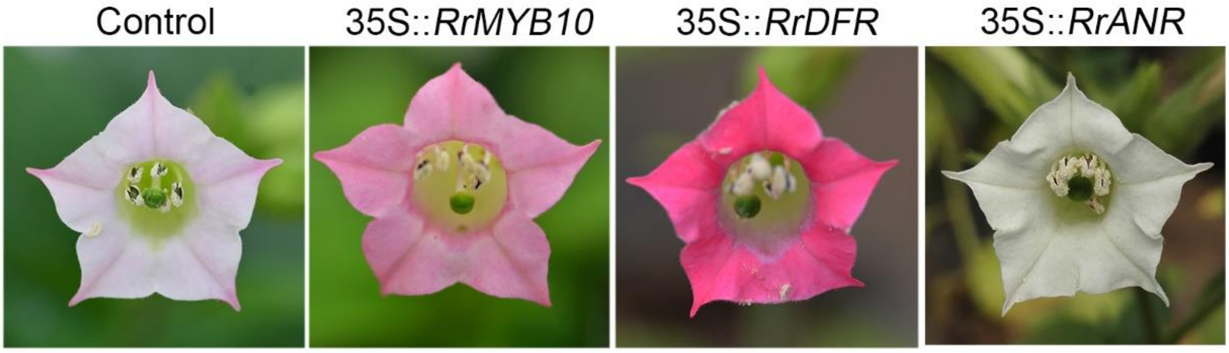

Figure S7: Phenotype of varied transgenic tobaccos used to perform RNA-Seq analysis. From left to right is the wide type, *RrMYB10*, *RrDFR* and *RrANR* transgenic tobaccos in turn.

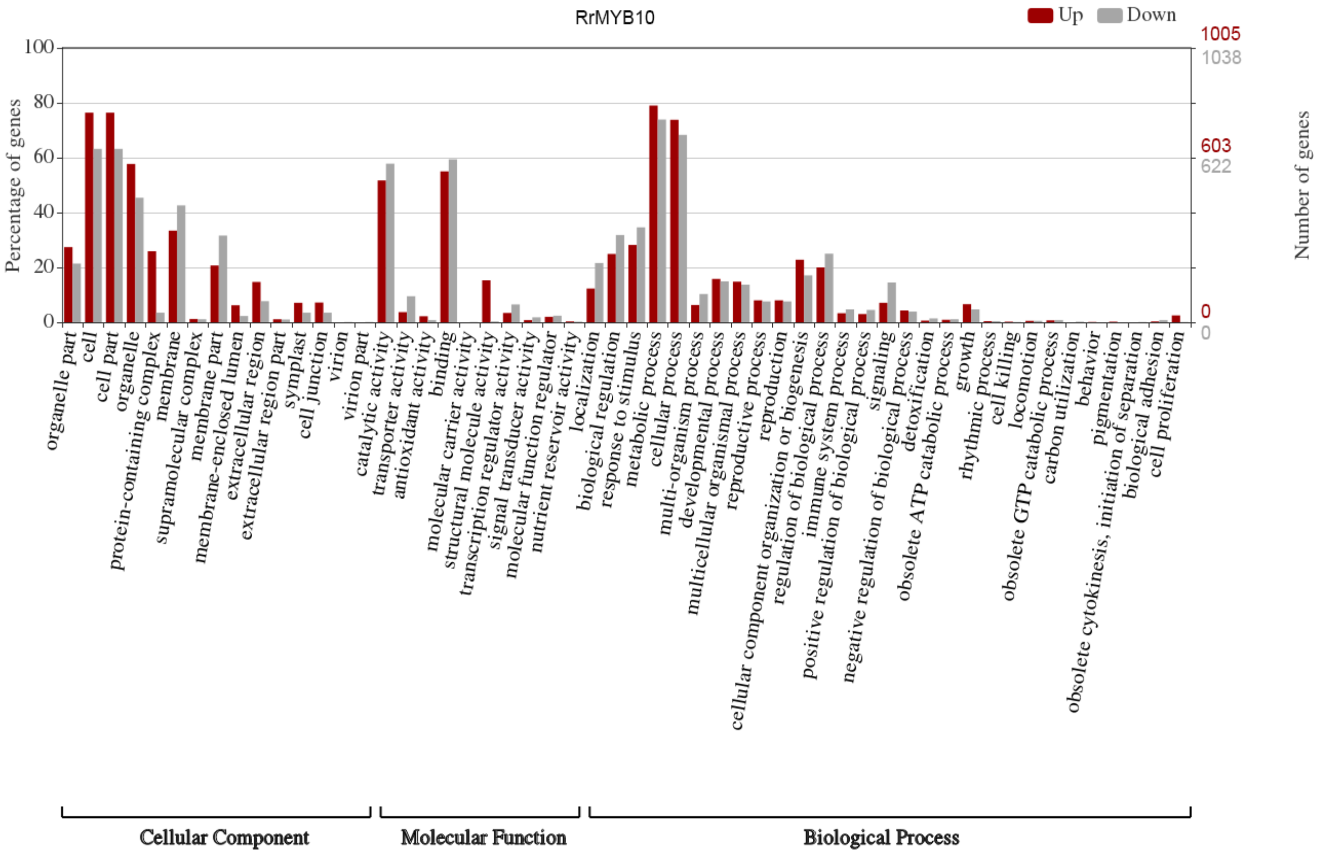

Figure S8: Histogram of gene ontology (GO) categorization for the differentially expressed genes between the wild type and *RrMYB10* transgenic tobacco. Unigenes were categorized into three main categories: ‘cellular component’, ‘molecular function’ and ‘biological process’. Left y-axis indicates the percentage of genes from each category.

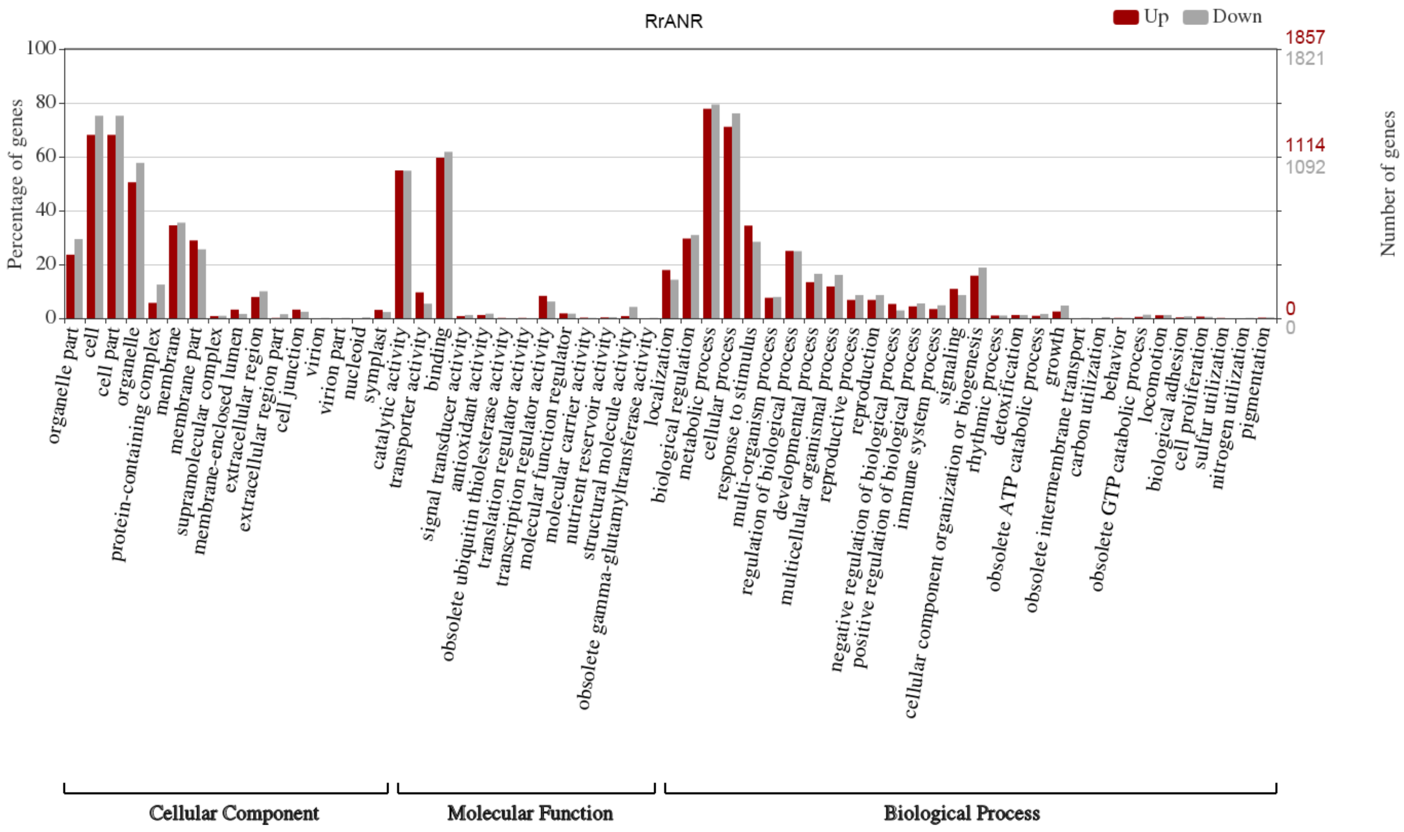

Figure S9: Histogram of gene ontology (GO) categorization for the differentially expressed genes between the wild type and *RrANR* transgenic tobacco lines. Graphs were constructed as described in Figure S8.

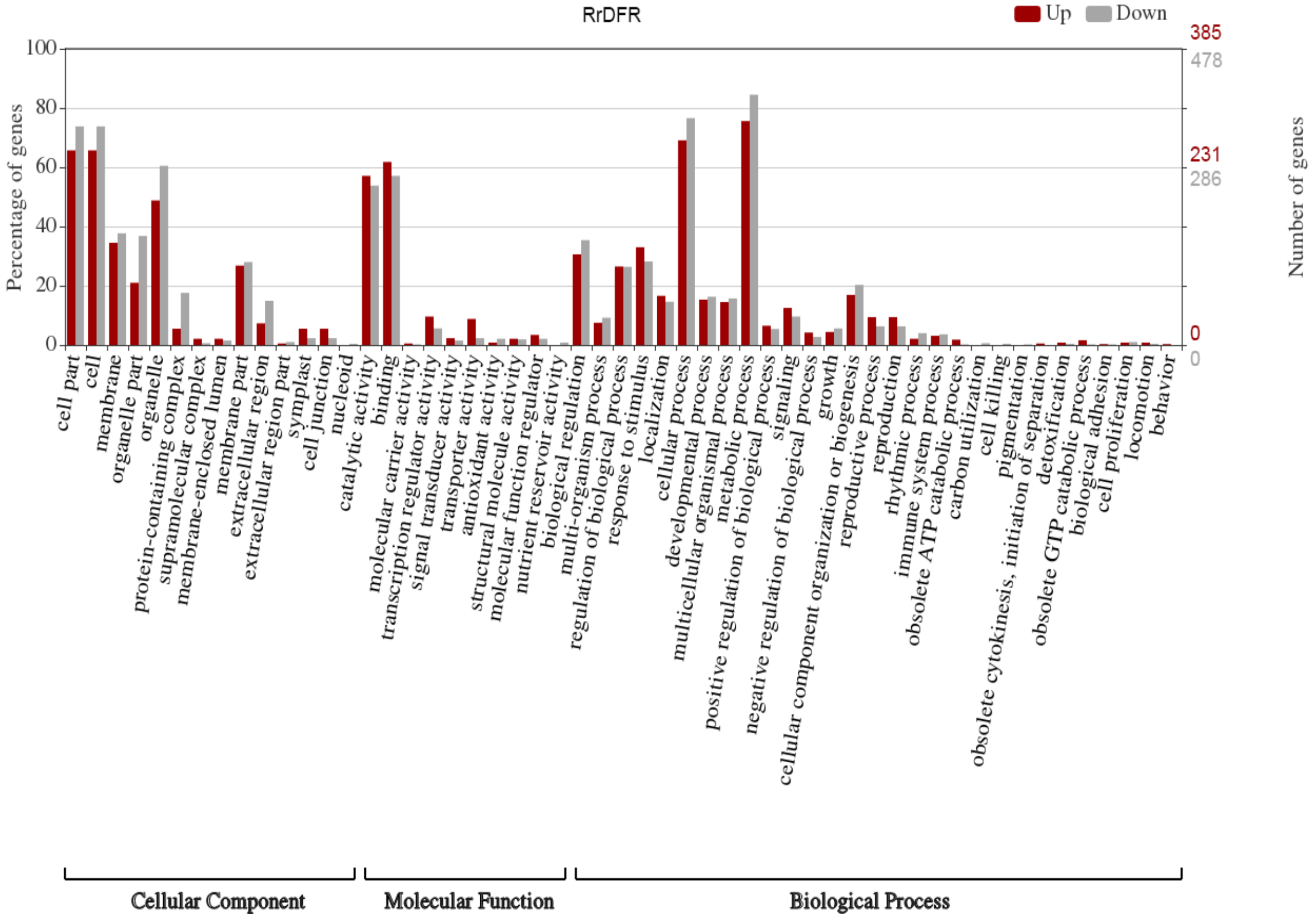

Figure S10: Histogram of gene ontology (GO) categorization for the differentially expressed genes between the wild type and *RrDFR* transgenic tobacco lines. Graphs were constructed as described in Figure S8.

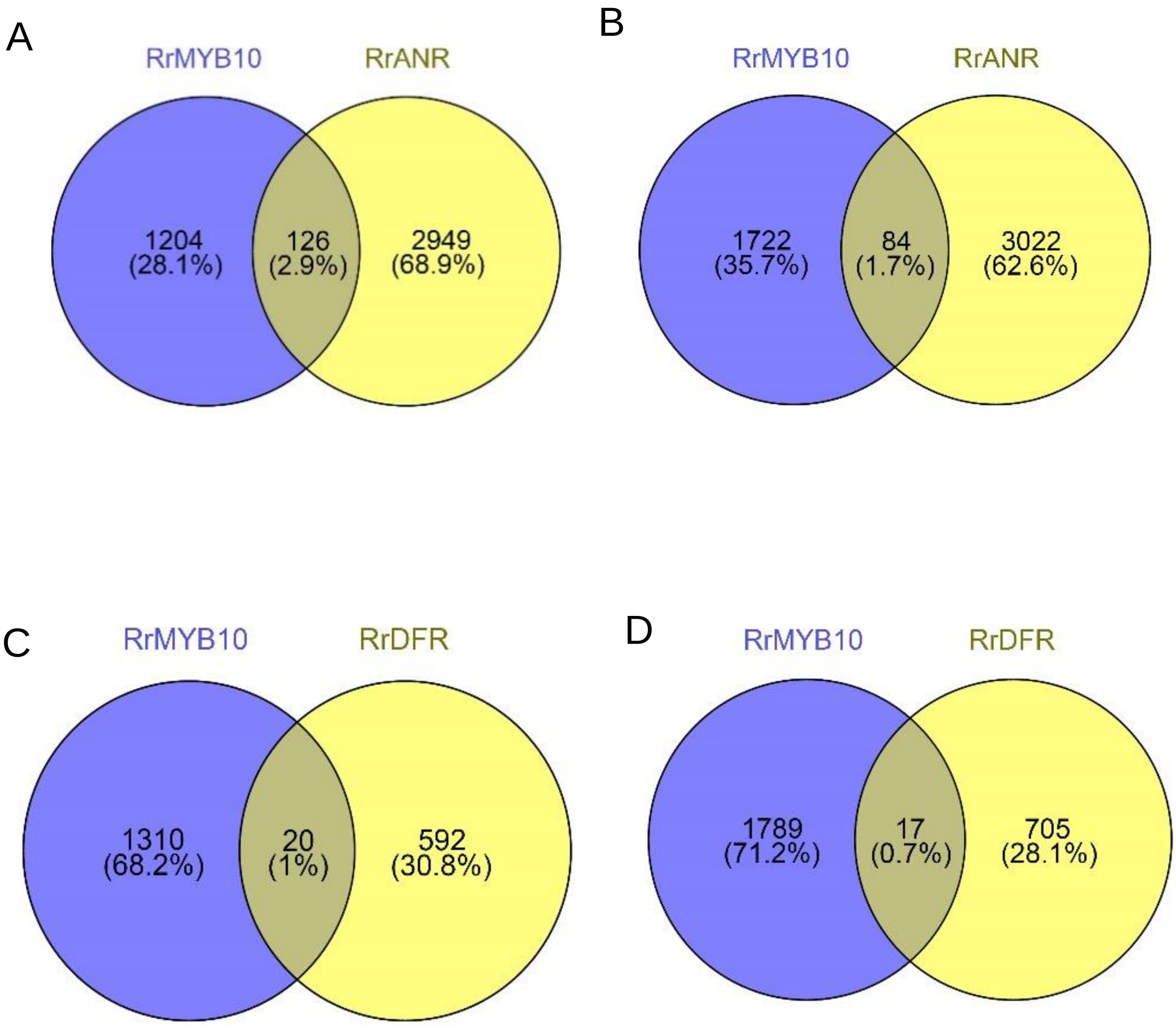

Figure S11: Comparisons of differentially expressed genes between *RrMYB10* with *RrANR*, and *RrMYB10* with *RrDFR* transgenic tobacco respectively. (A-B): Venn diagrams show the number of the upregulated genes (A) and downregulated genes (B) in *RrMYB10* and *RrANR* transgenic tobacco compared with wild type tobacco. (C-D): Venn diagrams show the number of the upregulated genes (C) and downregulated genes (D) in *RrMYB10* and *RrDFR* transgenic tobacco.

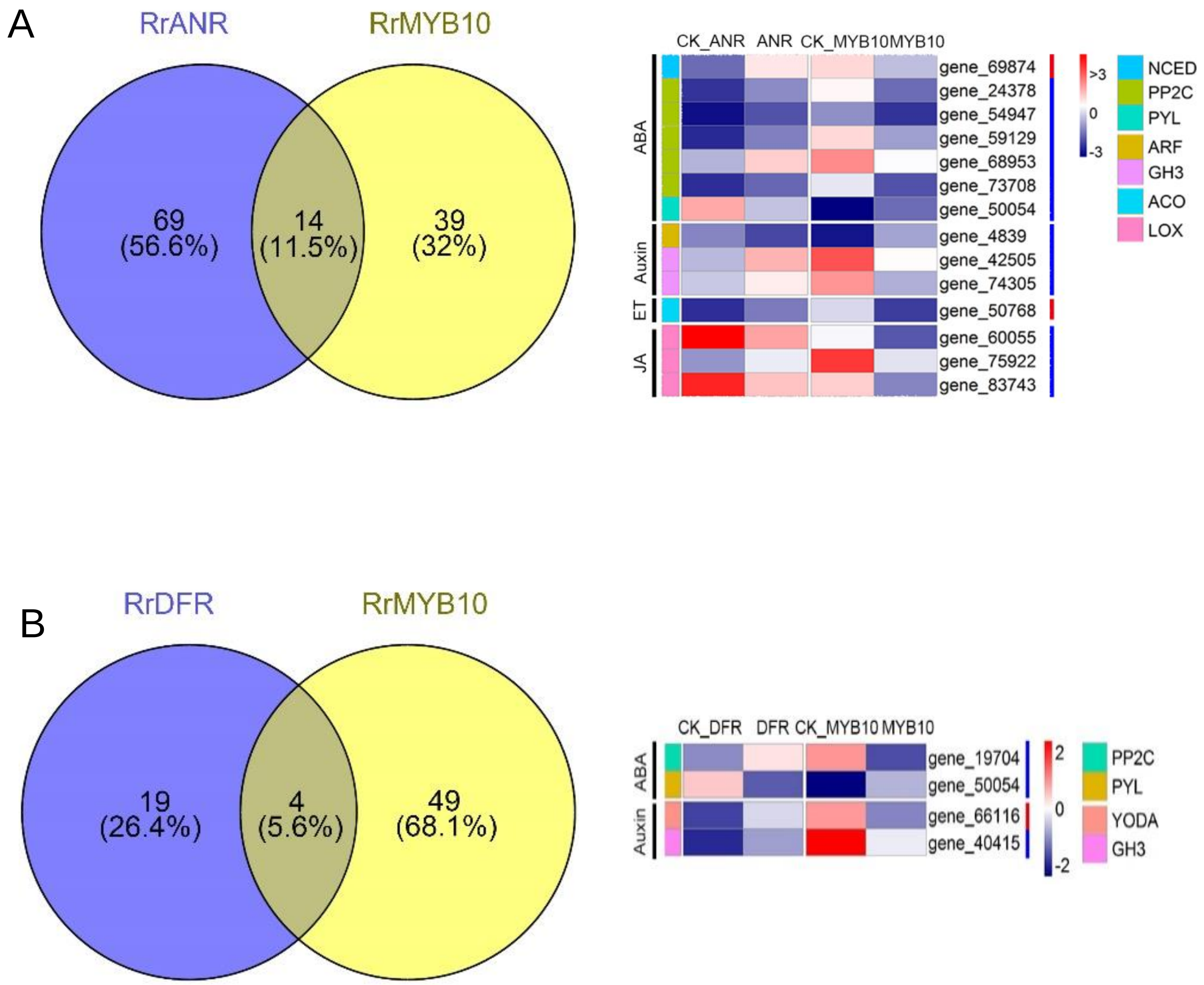

Figure S12: Common differentially expressed genes associated with hormone metabolism and signaling in varied transgenic tobaccos. (A) Venn diagrams show the number of DEGs associated with hormone metabolism and signaling in *RrMYB10* with *RrANR* transgenic tobacco (left) and a heatmap shows these hormone-related gene expressions (right). (B) Venn diagrams show the number of DEGs associated with hormone metabolism and signaling in *RrMYB10* with *RrDFR* transgenic tobacco (left) and a heatmap shows these hormone-related gene expressions (right). Red lines represent the genes involved in hormones anabolic metabolism and catabolism; blue lines represent the genes involved in hormone signaling pathways. ET: ethylene; JA: jasmonic acid; ABA: abscisic acid.

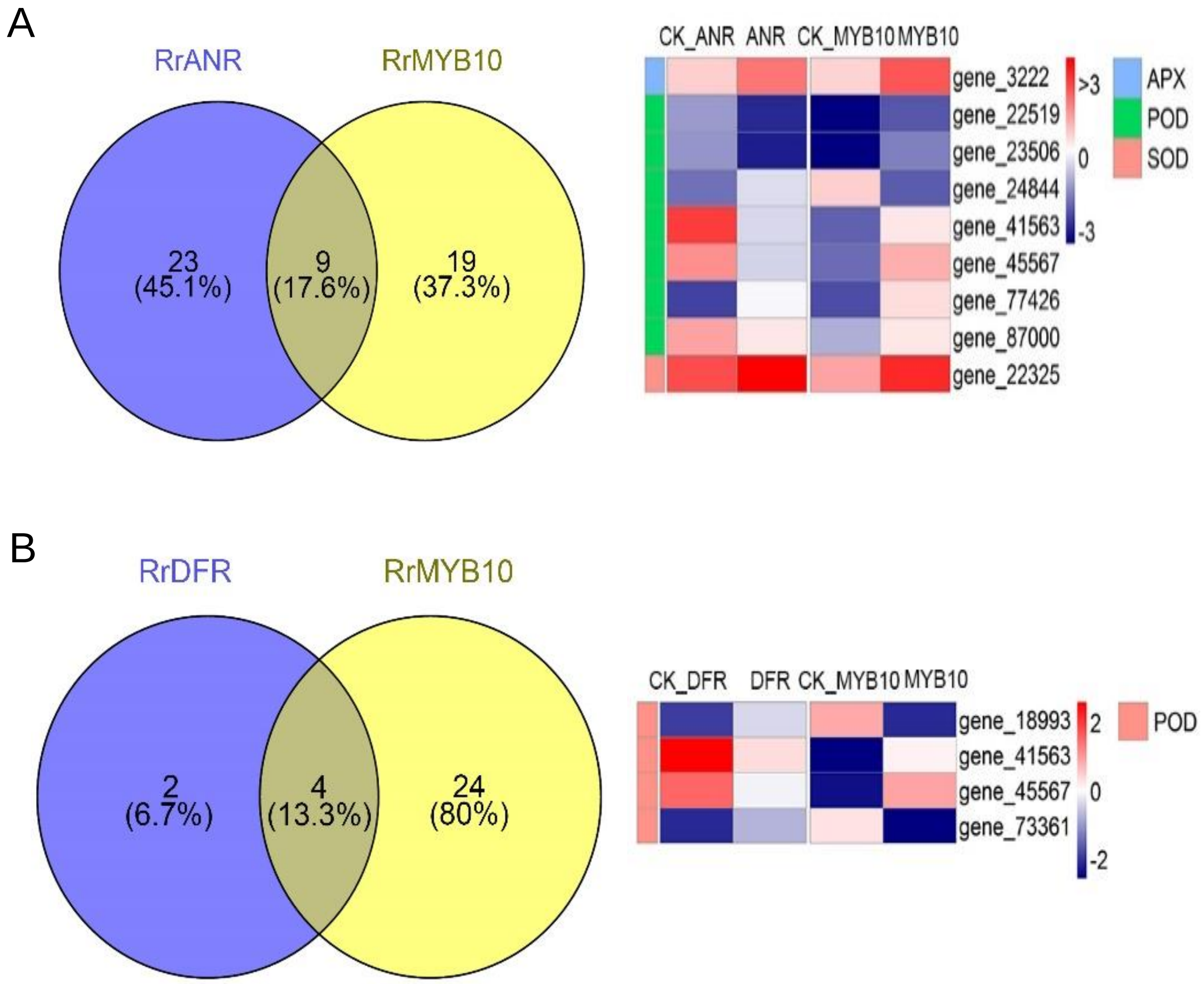

Figure S13: Common differentially expressed genes belonging to antioxidant-related genes in varied transgenic tobaccos. (A) Venn diagrams show the number of antioxidant-related genes in *RrMYB10* with *RrANR* transgenic tobacco (left) and a heatmap shows these hormone-related gene expressions (right). (B) Venn diagrams show the number of antioxidant-related genes in *RrMYB10* with *RrDFR* transgenic tobacco (left) and a heatmap shows these hormone-related gene expressions (right). APX: Ascorbate peroxidase; POD: peroxidase; SOD: superoxide dismutase.

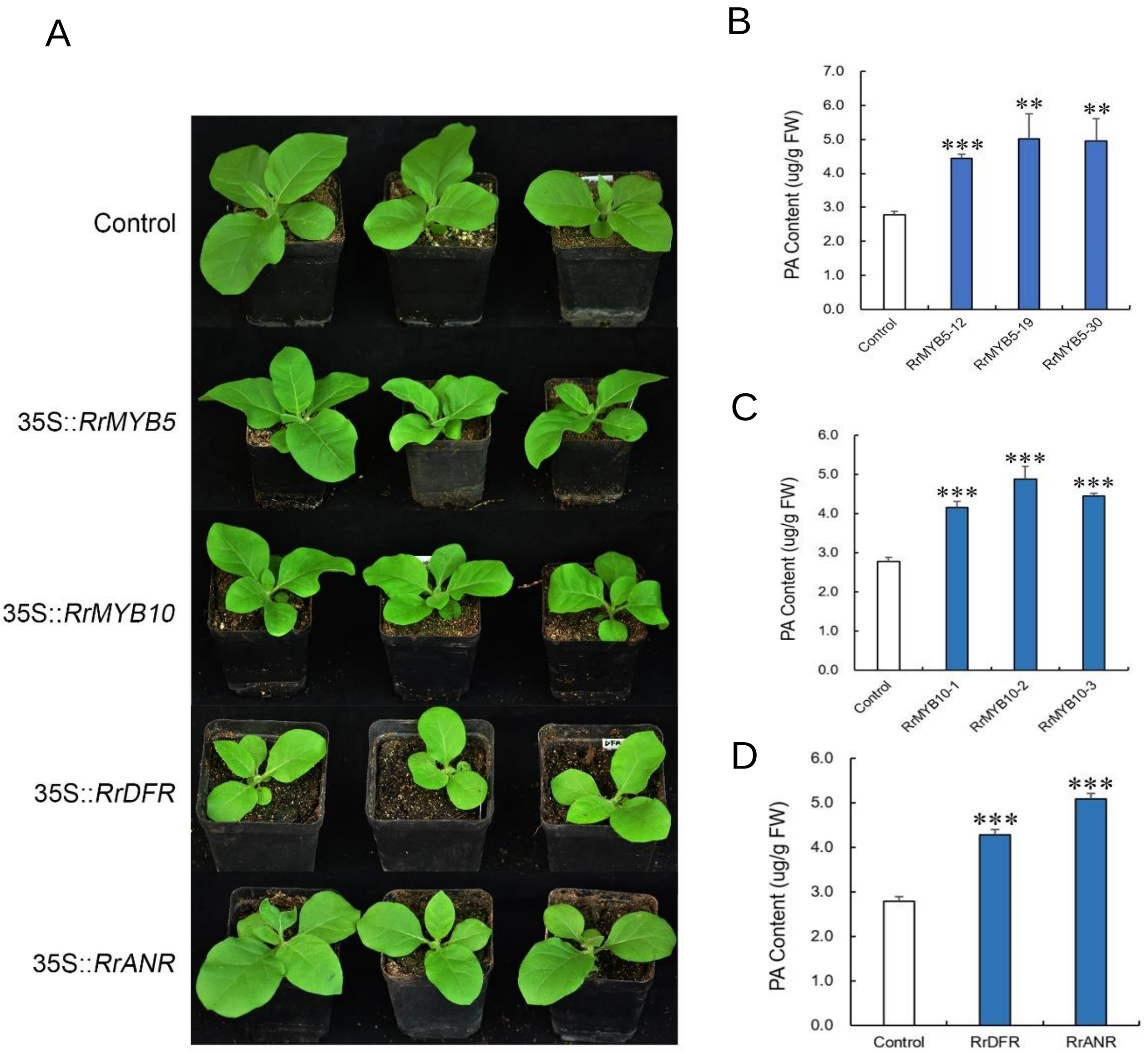

Figure S14: Phenotype and PA content measured in *RrMYB5*, *RrMYB10*, *RrANR* and *RrDFR* transgenic tobacco leaves. *(*A) Phenotypes of 6-week-old control and various transgenic tobacco plants. (B-D) PA levels in leaves of various 6-week-old transgenic tobacco plants. Data represent mean ± SE of three biological replicates (n = 3). Statistical significance was determined using Student's t test. (* P<0.05; ** P<0.01; *** P<0.001).

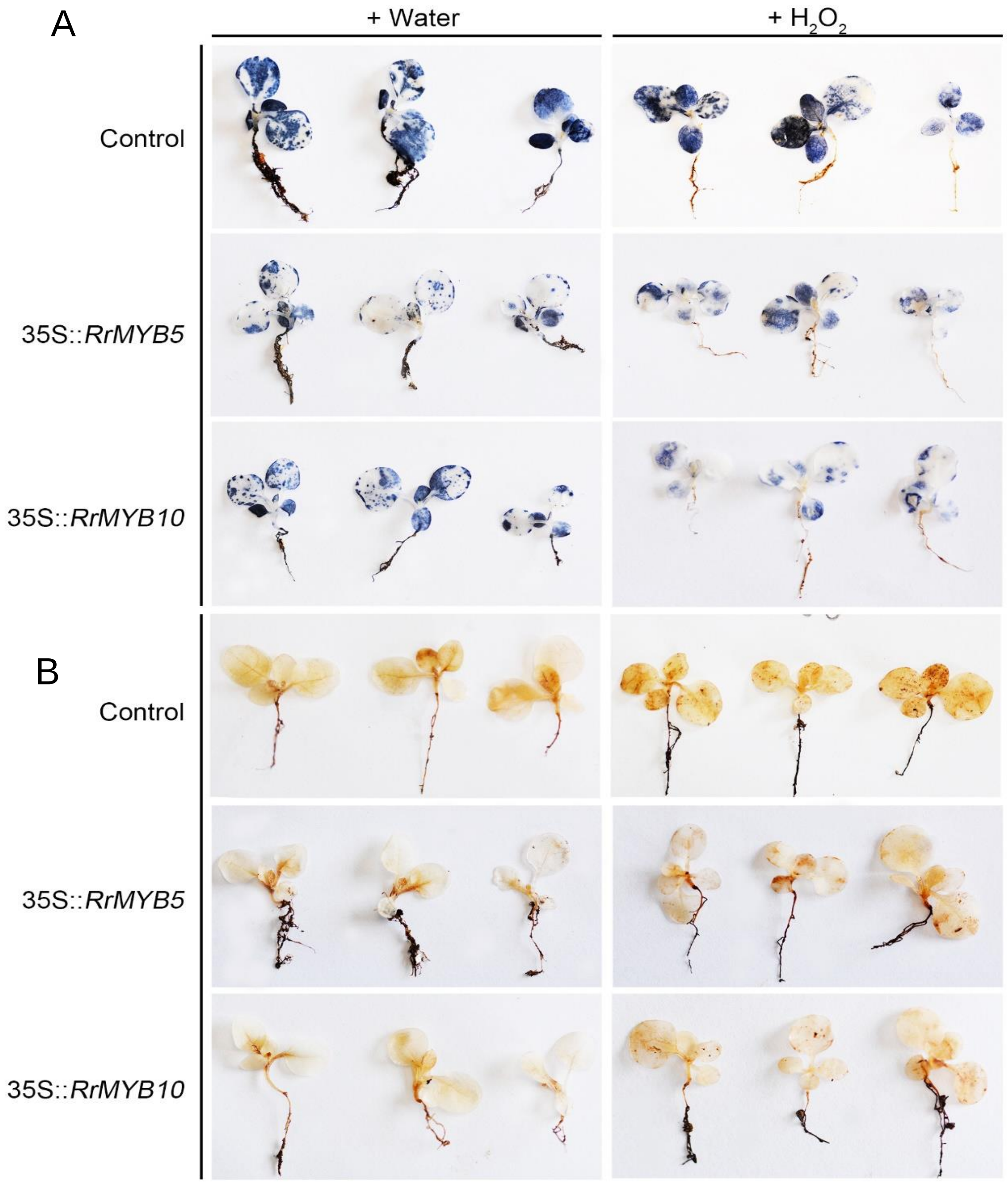

Figure S15: Representative photos of various 3-week-old transgenic tobacco plants harboring *35S::RrMYB5* and *35S::RrMYB10* showing staining with NBT (A) and DAB (B) after H_2O_2 treatment.

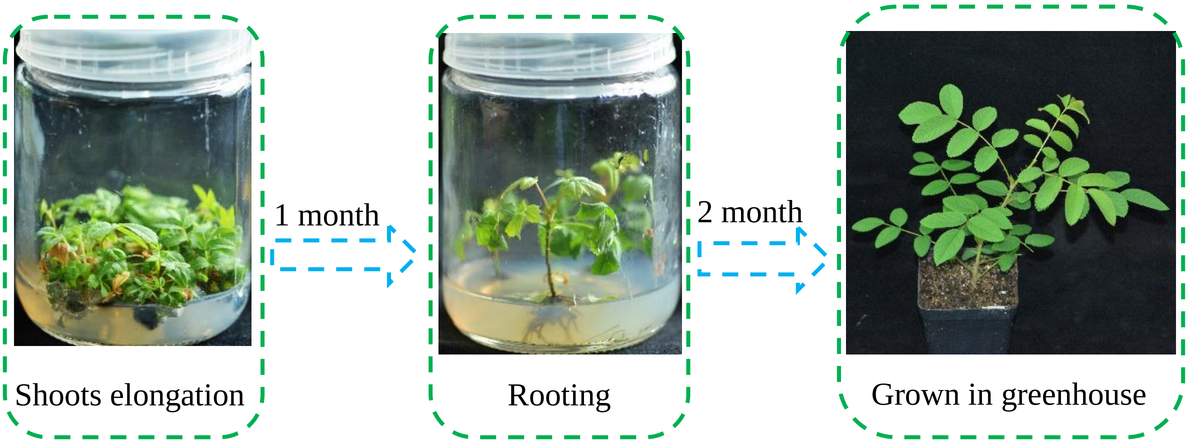

Figure S16: Plants tested in our experiments were propagated from one genotype by tissue culture. Plants of *R. rugosa* were propagated (left), shoots elongation and rooting (middle) and transferred to soil and grown in greenhouses (right).

Table S1. Stress-response related MYB genes previously reported in *Arabidopsis*.

Gene	Name	Abiotic stress										Biotic stress			References
		Salt	Wounding	Drought	Cold	Iron ion starvation	Phosphate starvation	Osmotic stress	Water deprivation	UV-B/High light	Oxidative stress	Bacterium	Insect	Fungus	
AT1G06180.1	MYB13	+												+	Qamar et al., 2010, Plant Journal
AT1G08810.1	MYB60							+	+						Galbiati et al., 2011, BMC Plant Biology
AT1G17950.1	MYB52	+		+					+						Park et al., 2011, Molecules & Cells
AT1G18570.1	MYB51	+	+									+	+		Cheong et al., 2002, Plant Physiology
AT1G18710.1	MYB47	+													Sottosanto et al., 2007, BMC Plant Biology
AT1G22640.1	MYB3	+	+												Cheong et al., 2002, Plant Physiology
AT1G35515.1	MYB8	+			+										Zhu et al., 2005, Plant Mol. Biol. Rep.
AT1G48000.1	MYB112	+								+					Lotkowska et al., 2015, Plant Physiology
AT1G56160.1	MYB72					+									Palmer et al., 2013, Plos Genetics
At1G56650.1	MYB75	+						+						+	Lee et al., 2016, Plant & Cell Physiology
AT1G68320.1	MYB62						+								Devaiah et al., 2009, Molecular Plant
AT1G74080.1	MYB122												+		Frerigmann et al., 2016, Molecular Plant
AT1G79180.1	MYB63									+					Zhong et al., 2009, Plant Signal Behav.
AT2G16720.1	MYB7	+													Fornalé et al., 2014, Plant & Cell Physiology
AT2G31180.1	MYB14	+			+										Chen et al., 2013, Plant Mol. Biol. Rep.
AT2G37630.1	MYB91	+										+			Nurmberg et al., 2007, PNAS
AT2G47190.1	MYB2	+						+	+						Baek et al., 2013, Plant Signal Behav.
At2G47460.1	MYB12	+		+						+					Wang et al., 2016, Molecular Genetics & Genomics
AT3G06490.1	MYB108	+												+	Mengiste et al., 2003, Plant Cell
AT3G12820.1	MYB10					+									Palmer et al., 2013, Plos Genetics

At3G13540.1	MYB5		+								+				Li et al., 2009, Plant Cell
AT3G23250.1	MYB15	+			+				+						Chezem et al., 2017, Plant Cell
AT3G47600.1	MYB94	+													Lee SB. et al., 2016, Plant & Cell Physiology
AT3G55730.1	MYB109	+													Harb, 2010, Virginia Polytechnic Institute & State University.
AT3G62610.1	MYB11									+					Stracke et al., 2010, New Phytologist
AT4G05100.1	MYB74	+													Xu et al., 2015, J. EXP. BOT.
AT4G21440.1	MYB102	+	+					+					+		Denekamp and Smeekens, 2003, Plant Physiology
AT4G34990.1	MYB32	+													Fornalé et al., 2014, Plant and Cell Physiology
AT4G37260.1	MYB73	+	+												Kim et al., 2013, Journal of Plant Physiology
AT4G38620.1	MYB4		+							+					Cheong et al., 2002, Plant Physiology
AT5G12870.1	MYB46													+	Ramírez et al., 2011, Plant Signal Behav.
AT5G15310.1	MYB16	+													Oshima and Mitsuda, 2013, Plant Signal Behav.
At5G35550.1	MYB123		+								+				Chen et al., 2012, Plant physiology
At5G49330.1	MYB111									+					Zhou et al., 2016, Plant Cell & Environment
AT5G62470.2	MYB96	+	+	+	+				+						Cheong et al., 2002, Plant Physiology
AT5G67300.1	MYB44	+	+						+					+	Jung et al., 2008, Plant Physiology

Table S2. Primers used to construct vectors.

Genus	Name	Primer sequence (5'-3')	Enzyme restriction site	Purpose
Rosa rugosa	RrMYB5-OE	F: GGGGTACCATGGGAAGGGCTCCTTGTTG	Kpn I	1
		R: GCGTCGACTCAGATCAACAGTGACTCAGCAAAC	Sal I	
	RrMYB10-OE	F: CGCGGATCCATGGGAAGAAGCAGCTCTCGT	BamH I	1
		R: GCGTCGACTCATAACTCTAAATGACCGATGTCG	Sal I	
	RrMYB5-SubL	F: GGGGTACCATGGGAAGGGCTCCTTGTTG	Kpn I	2
		R: GCGTCGACGATCAACAGTGACTCAGCAAAC	Sal I	
	RrMYB10-SubL	F: CGCGGATCCATGGGAAGAAGCAGCTCTCGT	BamH I	2
		R: GCGTCGACTAACTCTAAATGACCGATGTCGGCACC	Sal I	
	RrMYB5-AD	F:ATGGCCATGGAGGCCAGTGAATTCATGGGAAGGGCTCCTTGTTG	EcoR I	3
		R:ACGATTCATCTGCAGCTCGAGCTCTCAGATCAACAGTGACTCAGCAAAC	Sac I	
	RrMYB5-BD	F: CGGAATTCATGGGAAGGGCTCCTTGTTG	EcoR I	3
		R: CGGGATCCTCAGATCAACAGTGACTCAGCAAAC	BamH I	
	RrMYB5$_{1-242}$-BD	F: CGGAATTCATGGGAAGGGCTCCTTGTTG	EcoR I	3
		R: CGGGATCCTACAACATTAAGGATTATTCGAGAC	BamH I	
	RrMYB5$_{1-216}$-BD	F: CGGAATTCATGGGAAGGGCTCCTTGTTG	EcoR I	3
		R: CGGGATCCACCCCACCCTCTTTGAAATCAGACC	BamH I	
	RrMYB10-AD	F: CGCCATATGATGGGAAGAAGCAGCTCTCGT	Nde I	3
		R: CGCGGATCCTCATAACTCTAAATGACCGATGTCG	BamH I	
	RrMYB10-BD	F: CGCCATATGATGGGAAGAAGCAGCTCTCGT	Nde I	3
		R: GCGTCGACTCATAACTCTAAATGACCGATGTCG	Sal I	
	RrMYB5-SK	F:GGATCCCCCGGGCTGCAGGAATTCATGGGAAGGGCTCCTTGTTG	EcoR I	4
		R:ACCGGGCCCCCCCTCGAGGTCGACTCAGATCAACAGTGACTCAGCAAAC	Sal I	
	RrMYB10-SK	F:GGATCCCCCGGGCTGCAGGAATTCATGGGAAGAAGCAGCTCTCGT	EcoR I	4
		R:	Sal I	

		ACCGGGCCCCCCCTCGAGGTCGACTCATAACTCTAAATGACCGATGTCG		
	RrDFRpro-Luc	F: GGGGTACCTAGGCATAGGCAAATCCCATCA	Kpn I	4
		R: CCCTCGAGCTAAGGATAGAGGTGATGATCGGAG	Xho I	
	RrANRpro-Luc	F:CACTATAGGGCGAATTGGGTACCTTGATCTAACATTTTCAGTCCA	Kpn I	4
		R:GCCGCTCTAGAACTAGTGGATCCTTAAGTTCTTCTTCTGCTTTG	BamH I	
	RrLARpro-Luc	F: AACTGCAGCTTTGGTCGTGTTCTTGTTCTGC	Pst I	4
		R: CGGGATCCAAACAGAAGTCTTTCCTATTTATGA	BamH I	
	RrMYB10pro-Luc	F: CGGGGTACCAAAGGAGGGTCTCCGCTACCT	Kpn I	4
		R: CGCGTCGACCTCTCACTCCTGTTTCTGGTTTCTG	Sal I	
	RrMYB5pro-Luc	F:CACTATAGGGCGAATTGGGTACCTTCACTCTTTATTTTACGCAC	Kpn I	4
		R:GCCGCTCTAGAACTAGTGGATCCAGAGAAAAGGAAGGAAGAGACG	BamH I	
	RrMYB5-CLuc	F: GGGGTACCATGGGAAGGGCTCCTTGTTG	Kpn I	
		R: CGGGATCCGATCAACAGTGACTCAGCAAAC	BamH I	5
	RrMYB10-NLuc	F: CGGGATCCATGGGAAGAAGCAGCTCTCGT	BamH I	5
		R: GCGTCGACTAACTCTAAATGACCGATGTCG	Sal I	
Arabidopsis thaliana	AtEGL3-SK	F:GGATCCCCCGGGCTGCAGGAATTCATGGCAACCGGAGAAAACAGAACGG	EcoR I	4
		R:ACCGGGCCCCCCCTCGAGGTCGACTTAACATATCCATGCAACCCTTTGA	Sal I	
	AtTTG1-SK	F:GGATCCCCCGGGCTGCAGGAATTCATGGATAATTCAGCTCCAG	EcoR I	4
		R:ACCGGGCCCCCCCTCGAGGTCGACTCAAACTCTAAGGAGCTGCAT	Sal I	
	AtEGL3-BD	F: CGCCATATGATGGCAACCGGAGAAAACAGAACGG	Nde I	3
		R:CCGGAATTCTTAACATATCCATGCAACCCTTTGA	EcoR I	

Notes: The numbers in the column ‘Purpose’ represent: 1: Overexpression; 2: Subcellular localization; 3: Yeast two-hybrid assays; 4: Dual luciferase reporter assay; 5: Split luciferase complementation assay.

Table S3. Primers used for qRT-PCR and semiquantitative RT-PCR analysis.

Genus	Name	Primer sequence (5'-3')	Purpose
Rosa rugosa	DN12034	F: CACCAGAAGCACAAGGTCAGG	qRT-PCR
		R: AATCGCAACTCACCGTCTCG	
	DN15240 (RrMYB10)	F:AACAGCTCAAGACGGTCTAGAAACG	qRT-PCR and semi-RT-PCR
		R: CACTTCCACCAGTGGTCTGCAAGA	
	DN23499	F: TCCACAAGTTCCAAAGCCAGC	qRT-PCR
		R: CCCAACACTCCAAGCCCAT	
	DN26257	F: AACATCTCCCGCTCCGACCA	qRT-PCR
		R: TGAACTTACCGACTGGGTTTGAG	
	DN26493	F: CATTGCTAACCACGGCGAA	qRT-PCR
		R: GCGAGTCCTCCAGTAGTTTTTGAT	
	DN28996	F: GAACACCCACCTGAAGAAGAAACT	qRT-PCR
		R: TGGAGAGTGAGGGCTTTAAGTCTG	
	DN29300	F: CTTTACCAACAGCACCACCACA	qRT-PCR
		R:TGAAGAAGTCGTCCTCTAAACCAAG	
	DN30644	F: GCATGTCATTATGGCTCGCA	qRT-PCR
		R: TTGTCATTCATCGCCTCCTGT	
	DN32810	F: ACAAGCCCAAAAACGACGC	qRT-PCR
		R: TTTGATTCCCTAACGAGCCG	
	DN32830	F: CGTCAGGTCAACCAAAACAGTG	qRT-PCR
		R: CTAATCCCAATCCGCTTTACAACT	
	DN32923	F: TTTATGGTGAGCGGTCGGTG	qRT-PCR
		R: ATTCAAAACTCGGAAAACAGGG	
	DN33327 (RrMYB5)	F: ATCGCATAGTGCCCGAAAAGCCT	qRT-PCR and semi-RT-PCR
		R: CACCAACAGAGAAGACCACCCCA	
	DN34526	F: CCTTCTGATTTTGTGGGGTATTTC	qRT-PCR
		R: AGTTGGACCTGGAGTTTTGATTG	
	DN36867	F: GATTGCGGGGCGTTTACCT	qRT-PCR
		R: GTGTGGCAGTTGGAGCAGTTC	
	DN39667	F: GCCAAGTGGGGTAACAGGTG	qRT-PCR
		R: AAAGTCGGCATCCAGAAACATC	
	DN40669	F: TGTCCCTTTCGCTCCCTGGT	qRT-PCR
		R: CCTCCTTCTTTATCATCCCCTGC	
	DN40785	F: CTCCTGGTTCAGTCCCTTGTGG	qRT-PCR
		R: CCCTTAGAGCCTTCACCTTCG	
	DN44524	F: AGATGGTCTTTGATAGCGGGAA	qRT-PCR
		R: CGTGGATGAGTGATCTTGAACTTT	
	RrCHS	F: GCCTGAAACCCGAAAAGTTAGAA	qRT-PCR and semi-RT-PCR

		R: CAAAACCAAATAGGACACCCCAC	
	RrCHI	F: ATGCCATCTCTCCAAGAATCAAAT<br>R: AGAACACATCAAAAAGTGCCGAA	qRT-PCR and semi-RT-PCR
	RrF3H	F: AGGTTGTCCATAGCCACATTCCA<br>R: AGGTCCTTGCTCATCTTCTTCTTGT	qRT-PCR and semi-RT-PCR
	RrF3'H	F: ATCAAGCACGGTGGAATGGG<br>R: GGATGGGTGGAGTCGGAACG	qRT-PCR and semi-RT-PCR
	RrDFR	F: CAAGGGCATTGAGGAGAACTTGC<br>R: CCTGTGACTTTGACACGGACGA	qRT-PCR and semi-RT-PCR
	RrFLS	F: TGGAGGGATACGGAACATTTTTA<br>R: CACCACCTTGTGTAGATTCTTTGC	qRT-PCR and semi-RT-PCR
	RrLAR	F: AAGGTGAGGCGTGCGATTGAGAA<br>R: CGGAACAACCTCTGAAGGATGCT	qRT-PCR and semi-RT-PCR
	RrANR	F: AATGGGTTGGTCTGATGTTGAGT<br>R: ATTTCCTGTAATTAAAGATGTGGCG	qRT-PCR and semi-RT-PCR
	RrANS	F: GGGATTAGAAGAAGGGAGGCTGG<br>R: CGGCTGAGGGCATTTTGGGTAGT	qRT-PCR and semi-RT-PCR
	RrGAPDH	F: TGAAGGGTGGTGCCAAGAA<br>R: AAGGGGAGCAAGACAGTTGG	qRT-PCR and semi-RT-PCR
Rosa hybrida	RhMYB5	F: ATCGCATAGTGCCCGAAAAGCCT<br>R: CACCAACAGAGAAGACCACCCCA	qRT-PCR and semi-RT-PCR
	RhMYB10	F:AACAGCTCAAGACGGTCTAGAAACG<br>R: CACTTCCACCAGTGGTCTGCAAGA	qRT-PCR and semi-RT-PCR
	RhCHS	F: GCCTGAAACCCGAAAAGTTAGAA<br>R: CAAAACCAAATAGGACACCCCAC	qRT-PCR
	RhCHI	F: ATGCCATCTCTCCAAGAATCAAAT<br>R: AGAACACATCAAAAAGTGCCGAA	qRT-PCR
	RhF3H	F: AGGTTGTCCATAGCCACATTCCA<br>R: AGGTCCTTGCTCATCTTCTTCTTGT	qRT-PCR
	RhF3'H	F: ATCAAGCACGGTGGAATGGG<br>R: GGATGGGTGGAGTCGGAACG	qRT-PCR
	RhDFR	F: CAAGGGCATTGAGGAGAACTTGC<br>R: CCTGTGACTTTGACACGGACGA	qRT-PCR

	RhFLS	F: TGGAGGGATACGGAACATTTTTA<br>R: CACCACCTTGTGTAGATTCTTTGC	qRT-PCR
	RhLAR	F: AAGGTGAGGCGTGCGATTGAGAA<br>R: CGGAACAACCTCTGAAGGATGCT	qRT-PCR
	RhANR	F: AATGGGTTGGTCTGATGTTGAGT<br>R: ATTTCCTGTAATTAAAGATGTGGCG	qRT-PCR
	RhANS	F: GGGATTAGAAGAAGGGAGGCTGG<br>R: CGGCTGAGGGCATTTTGGGTAGT	qRT-PCR
	RhGAPDH	F: TGAAGGGTGGTGCCAAGAA<br>R: AAGGGGAGCAAGACAGTTGG	qRT-PCR
Nicotiana tabacum	NtCHS	F:AAGCAAGAGAAACTAAAGGCTACAAG<br>R: AAATCCAAAAAGCACACCCCAT	qRT-PCR
	NtCHI	F: CGGGTGCCTCCATTCTTTTTACT<br>R: CCTGACACTCTTTCGGCGATACTAC	qRT-PCR
	NtF3H	F:CCAGACAAACCAGATGGATGGATAG<br>R: CAAGGGTAAGGTCGGGCTGTG	qRT-PCR
	NtF3'H	F: TGGCTATTTCATTCCAAAAGGCTCA<br>R: CTTCAAAGTCATTTCCTCGCACATC	qRT-PCR
	NtFLS	F: CTTGAAGGGAAAAGGGGTTGG<br>R: CGCAACTTCTCGCAGCCTCT	qRT-PCR
	NtDFR	F: GCAGTTGCTTCCCTTTTCTACC<br>R: TTCCCCATTGGTTGACTTTCC	qRT-PCR
	NtLAR	F: TCAATGGTGCGAAAGGACTC<br>R: TGCTGCAGAGAATATCAACC	qRT-PCR
	NtANR	F: CATTTGACTTTCCCAAACGC<br>R: ATTGGGCTTTTGAGTTGTGC	qRT-PCR
	NtANS	F: GTGCCTGGGTTACAACTTTTCTATG<br>R: CATTGCTTAGGATTTCAAGGGTGTC	qRT-PCR
	NtUFGT	F: ATGTTGAAGGGCTAAAAGAAAGAGC<br>R: CAAGTCCCAGCTGATACATATTCCC	qRT-PCR
	NtEF-1-a	F: TGGTTGTGACTTTTGGTCCCA<br>R: ACAAACCCACGCTTGAGATCC	qRT-PCR
Arabidopsis thaliana	AtEGL3	F: GACGACGATGTTCATTACCAAGG<br>R: TTATCATCTTCTGCGATTTCTCTCC	semi-RT-PCR

Table S4-attached separately

Table S4. Amino acid sequences from strawberry, *Arabidopsis*, and *R. rugosa* MYB transcription factors and used for Phylogenetic analysis.

Table S5. Summary of sample short reads from SGS sequencing of *Rosa rugosa* after clearing.

Sample	Read Length	Clean reads	Clean Bases(G)	Q20 Rate(%)	Q30 Rate(%)
RrBBB1_1	150	13312048	3.8866	0.9939	0.9158
RrBBB2_1	150	14229752	4.1280	0.9928	0.9123
RrBBB3_1	150	13842391	4.0157	0.9938	0.9201
RrBBH1_1	150	11482673	3.3525	0.9917	0.8982
RrBBH2_1	150	11054835	3.2276	0.9926	0.9018
RrBBH4_1	150	13013021	3.7993	0.9911	0.88646

Table S6. Summary of transcripts assembled from *Rosa Rugosa* short-read data using Trinity.

Sample	Total length	Total transcripts	Average length	N50 length	Max length	Min length
Trinity	196257693	298,735	657	980	11182	201

Table S7. Summary of sample short reads from SGS sequencing of tobacco after clearing.

Sample	Read Length	Clean reads	Clean Bases(G)	Q20 Rate(%)	Q30 Rate(%)
CK_MYB10_1_1	150	11948340	3.9573	0.9947	0.9045
CK_MYB10_2_1	150	11926746	3.9502	0.9946	0.8972
CK_MYB10_3_1	150	11806423	3.9103	0.9948	0.9045
MYB10_1_1	150	9227398	3.0561	0.9951	0.9656
MYB10_2_1	150	11207442	3.7119	0.9958	0.9641
MYB10_3_1	150	9861631	3.2662	0.9963	0.9608
CK_DFR_1_1	150	9777333	2.7726	0.9960	0.9611
CK_DFR_2_1	150	7049898	1.9992	0.9952	0.9623
CK_DFR_3_1	150	9309409	2.6399	0.9959	0.9621
DFR_1_1	150	6711522	1.9032	0.9960	0.9195
DFR_2_1	150	9295064	2.6359	0.9958	0.9238
DFR_3_1	150	11408397	3.2351	0.9963	0.9237

Table S8-attached separately

Table S8. Up- and downregulated genes in transgenic tobacco overexpressing *RrMYB10*.

Table S9-attached separately

Table S9. Up- and downregulated genes in transgenic tobacco overexpressing *RrDFR*.

Table S10-attached separately

Table S10. Up- and downregulated genes in transgenic tobacco overexpressing *RrANR*.
