# Supplementary material for: RrMYB5‐ and RrMYB10‐regulated flavonoid biosynthesis plays a pivotal role in feedback loop responding to wounding and oxidation in Rosa rugosa
Source: Plant Biotechnol J. 2019 Apr 14;17(11):2078–95. doi: 10.1111/pbi.13123 (PMC6790370; doi:10.1111/pbi.13123)
Supplement: Supplementary file 2 — Table S4 Amino acid sequences from strawberry, Arabidopsis, and R. rugosa MYB transcription factors and used for Phylogenetic analysis. [file PBI-17-2078-s003.docx]

Table S4. Amino acid sequences from strawberry, *Arabidopsis*, and *R. rugosa* MYB transcription factors and used for Phylogenetic analysis.

>AT1G06180.1

KGPWSAEEDRILINYISLHGHPNWRALPKLAGLLRCGKSCRLRWINYLRPDIKRGNFTPHEEDTIISLHQLLGNRWSAIAAKLPGRTDNEIKNVWHTHLKKR

>AT1G08810.1

KGPWTPEEDIILVSYIQEHGPGNWRSVPTNTGLLRCSKSCRLRWTNYLRPGIKRGNFTPHEEGMIIHLQALLGNKWASIASYLPQRTDNDIKNYWNTHLKKK

>AT1G17950.1

RGHWRPAEDEKLRELVEQFGPHNWNAIAQKLSGRSGKSCRLRWFNQLDPRINRNPFTEEEEERLLASHRIHGNRWSVIARFFPGRTDNAVKNHWHVIMARR

>AT1G18570.1

KGAWTPEEDQKLLSYLNRHGEGGWRTLPEKAGLKRCGKSCRLRWANYLRPDIKRGEFTEDEERSIISLHALHGNKWSAIARGLPGRTDNEIKNYWNTHIKKR

>AT1G18710.1

KGEWTAEEDQKLGAYINEHGVCDWRSLPKRAGLQRCGKSCRLRWLNYLKPGIRRGKFTPQEEEEIIQLHAVLGNRWAAMAKKMQNRTDNDIKNHWNSCLKKR

>AT1G22640.1

KGAWTKEEDQLLVDYIRKHGEGCWRSLPRAAGLQRCGKSCRLRWMNYLRPDLKRGNFTEEEDELIIKLHSLLGNKWSLIAGRLPGRTDNEIKNYWNTHIKRK

>AT1G35515.1

KGAWTKEEDQRLIDYIRNHGEGSWRSLPKSVGLLRCGKSCRLRWINYLRPDLKRGNFTDGEEQIIVKLHSLFGNKWSLIAGKLPGRTDNEIKNYWNTHIKRK

>AT1G48000.1

RGPWTVEEDMKLVSYISLHGEGRWNSLSRSAGLNRTGKSCRLRWLNYLRPDIRRGDISLQEQFIILELHSRWGNRWSKIAQHLPGRTDNEIKNYWRTRVQKH

>AT1G56160.1

RGPWSPQEDLTLITFIQKHGHQNWRSLPKLAGLLRCGKSCRLRWINYLRPDVKRGNFSKKEEDAIIHYHQTLGNKWSKIASFLPGRTDNEIKNVWNTHLKKR

>AT1G56650.1

KGAWTTEEDSLLRQCINKYGEGKWHQVPVRAGLNRCRKSCRLRWLNYLKPSIKRGKLSSDEVDLLLRLHRLLGNRWSLIAGRLPGRTANDVKNYWNTHLSKK

>AT1G68320.1

RGPWTLEEDTLLTNYILHNGEGRWNHVAKCAGLKRTGKSCRLRWLNYLKPDIRRGNLTPQEQLLILELHSKWGNRWSKIAQYLPGRTDNEIKNYWRTRVQKQ

>AT1G74080.1

KGAWTQEEDQKLIAYVQRHGEGGWRTLPDKAGLKRCGKSCRLRWANYLRPDIKRGEFSQDEEDSIINLHAIHGNKWSAIARKIPRRTDNEIKNHWNTHIKKC

>AT1G79180.1

RGPWSPEEDIKLISFIQKFGHENWRSLPKQSGLLRCGKSCRLRWINYLRPDLKRGNFTSEEEETIIKLHHNYGNKWSKIASQLPGRTDNEIKNVWHTHLKKR

>AT2G16720.1

KGAWTKEEDERLVSYIKSHGEGCWRSLPRAAGLLRCGKSCRLRWINYLRPDLKRGNFTHDEDELIIKLHSLLGNKWSLIAARLPGRTDNEIKNYWNTHIKRK

>AT2G31180.1

RGPWTPEEDQILINYIHLYGHSNWRALPKHAGLLRCGKSCRLRWINYLRPDIKRGNFTPQEEQTIINLHESLGNRWSAIAAKLPGRTDNEIKNVWHTHLKKR

>AT2G37630.1

RQRWSGEEDALLRAYVRQFGPREWHLVSERMNKPLNRDAKSCLERWKNYLKPGIKKGSLTEEEQRLVIRLQEKHGNKWKKIAAEVPGRTAKRLGKWWEVFKEKQ

>AT2G47190.1

KGPWTEEEDAILVNFVSIHGDARWNHIARSSGLKRTGKSCRLRWLNYLRPDVRRGNITLEEQFMILKLHSLWGNRWSKIAQYLPGRTDNEIKNYWRTRVQKQ

>AT2G47460.1

RGRWTAEEDQILSNYIQSNGEGSWRSLPKNAGLKRCGKSCRLRWINYLRSDLKRGNITPEEEELVVKLHSTLGNRWSLIAGHLPGRTDNEIKNYWNSHLSRK

>AT3G06490.1

RGPWTAEEDFKLMNYIATNGEGRWNSLSRCAGLQRTGKSCRLRWLNYLRPDVRRGNITLEEQLLILELHSRWGNRWSKIAQYLPGRTDNEIKNYWRTRVQKH

>AT3G12820.1

RGPWSDEESERLRSFILKNGHQNWRSLPKLAGLMRCGKSCRLRWINYLRPGLKRGNFTKEEEDTIIHLHQAYGNKWSKIASNFPGRTDNEIKNVWNTHLKKR

>AT3G13540.1

RGPWTVEEDEILVSFIKKEGEGRWRSLPKRAGLLRCGKSCRLRWMNYLRPSVKRGGITSDEEDLILRLHRLLGNRWSLIAGRIPGRTDNEIKNYWNTHLRKK

>AT3G23250.1

RGPWTPEEDQILVSFILNHGHSNWRALPKQAGLLRCGKSCRLRWMNYLKPDIKRGNFTKEEEDAIISLHQILGNRWSAIAAKLPGRTDNEIKNVWHTHLKKR

>AT3G47600.1

KGPWTPEEDIILVSYIQEHGPGNWRSVPTHTGLRRCSKSCRLRWTNYLRPGIKRGNFTEHEEKMILHLQALLGNRWAAIASYLPERTDNDIKNYWNTHLKKK

>AT3G55730.1

KGPWSTEEDAVLTKLVRKLGPRNWSLIARGIPGRSGKSCRLRWCNQLDPCLKRKPFSDEEDRMIISAHAVHGNKWAVIAKLLTGRTDNAIKNHWNSTLRRK

>AT3G62610.1

KGRWTAEEDRTLSDYIQSNGEGSWRSLPKNAGLKRCGKSCRLRWINYLRSDIKRGNITPEEEDVIVKLHSTLGTRWSTIASNLPGRTDNEIKNYWNSHLSRK

>AT4G05100.1

KGPWTPEEDQKLIDYINIHGYGNWRTLPKNAGLQRCGKSCRLRWTNYLRPDIKRGRFSFEEEETIIQLHSIMGNKWSAIAARLPGRTDNEIKNYWNTHIRKR

>AT4G21440.1

KGPWTSEEDQKLVDYIQKHGYGNWRTLPKNAGLQRCGKSCRLRWTNYLRPDIKRGRFSFEEEETIIQLHSFLGNKWSAIAARLPGRTDNEIKNFWNTHIRKK

>AT4G34990.1

KGAWTKEEDDKLISYIKAHGEGCWRSLPRSAGLQRCGKSCRLRWINYLRPDLKRGNFTLEEDDLIIKLHSLLGNKWSLIATRLPGRTDNEIKNYWNTHVKRK

>AT4G37260.1

KGPWSPEEDDLLQRLVQKHGPRNWSLISKSIPGRSGKSCRLRWCNQLSPEVEHRAFSQEEDETIIRAHARFGNKWATISRLLNGRTDNAIKNHWNSTLKRK

>AT4G38620.1

KGAWTKEEDERLVAYIKAHGEGCWRSLPKAAGLLRCGKSCRLRWINYLRPDLKRGNFTEEEDELIIKLHSLLGNKWSLIAGRLPGRTDNEIKNYWNTHIRRK

>AT5G12870.1

KGLWSPEEDSKLMQYMLSNGQGCWSDVAKNAGLQRCGKSCRLRWINYLRPDLKRGAFSPQEEDLIIRFHSILGNRWSQIAARLPGRTDNEIKNFWNSTIKKR

>AT5G15310.1

KGPWTPEEDQKLLAYIEEHGHGSWRSLPEKAGLHRCGKSCRLRWTNYLRPDIKRGKFNLQEEQTIIQLHALLGNRWSAIATHLPKRTDNEIKNYWNTHLKKR

>AT5G35550.1

RGAWTDHEDKILRDYITTHGEGKWSTLPNQAGLKRCGKSCRLRWKNYLRPGIKRGNISSDEEELIIRLHNLLGNRWSLIAGRLPGRTDNEIKNHWNSNLRKR

>AT5G49330.1

RGRWTAEEDEILTKYIQTNGEGSWRSLPKKAGLLRCGKSCRLRWINYLRRDLKRGNITSDEEEIIVKLHSLLGNRWSLIATHLPGRTDNEIKNYWNSHLSRK

>AT5G62470.2

KGPWTPEEDIILVSYIQEHGPGNWRSVPTHTGLRRCSKSCRLRWTNYLRPGIKRGNFTEHEEKTIVHLQALLGNRWAAIASYLPERTDNDIKNYWNTHLKKK

>AT5G67300.1

KGPWSPEEDEQLRRLVVKYGPRNWTVISKSIPGRSGKSCRLRWCNQLSPQVEHRPFSAEEDETIARAHAQFGNKWATIARLLNGRTDNAVKNHWNSTLKRK

>mrna00185.1v1.0hybrid

KGPWSPEEDEALQRLVQSYGPRNWSLISKSIPGRSGKSCRLRWCNQLSPEVEHRPFTPEEDDTIIRAHARFGNKWATISRLLNGRTDNAIKNHWNSTLKRK

>mrna01013.1v1.0hybrid

RGHWRPAEDERLRQLVEQYGAQNWNSIAEKLQGRSGKSCRLRWFNQLDPRINRRPFSEEEEERLLAAHRIHGNKWAMIARLFPGRTDNAVKNHWHVIMARR

>mrna01992.1v1.0hybrid

KGPWTSEEDVILVNYIQEHGSGNWRNLPKNAGLQRCGKSCRLRWTNYLRPDIKRGRFSFEEEETIIQLHSILGNKWSAIAARLPGRTDNEIKNYWNTHIRKR

>mrna01997.1v1.0hybrid

KGAWDAEEDDKLRSYIQSYGHWNWRQLPKYAGISRCGKSCRLRWKNYLQPGVKHGDYTEEEEALIIKLHEQLGNRWSMIASKLPGRTDNEIKNYWHTHMKKK

>mrna02214.1v1.0hybrid

KGPWTTEEDQKLVDYINRNGHGSWRALPKLAGLNRCGKSCRLRWTNYLRPDIKRGKFSEEEERVIINLHAVVGNKWSKIATHLPGRTDNEIKNYWNTHLRKK

>mrna03321.1v1.0hybrid

KGPWTATEDQILMDYVRKHGEGNWNSVQRNSGLNRCGKSCRLRWANHLRPNLKKGAFTPEEERLILELHAKYGNKWARMASQLPGRTDNEIKNYWNTRVKRR

>mrna03712.1v1.0hybrid

KGPWTLDEDTLLIHYIENHGEGHWNALAKCAGLKRTGKSCRLRWLNYLKPDIKRGNLTPQEQLLILELHSKWGNRWSKIAQHLPGRTDNEIKNYWRTRVQKQ

>mrna03817.1v1.0hybrid

KGPWTPEEDIILVSYIQEHGPGNWRAVPTNTGLLRCSKSCRLRWTNYLRPGIKRGNFTDHEEKMIIHLQALLGNRWAAIASYLPQRTDNDIKNYWNTHLKKK

>mrna04423.1v1.0hybrid

KGPWTPEEDQKLLAYIEEHGHGSWRALPIKAGLQRCGKSCRLRWTNYLRPDIKRGKFSLQEEQTIIQLHALLGNRWSAIATHLPKRTDNEIKNYWNTHLKKR

>mrna05018.1v1.0hybrid

RGPWTVEEDLALMNYIANHGEGRWNSLARCAGLKRTGKSCRLRWLNYLRPDVRRGNITLEEQLLILELHSRWGNRWSKIAQHLPGRTDNEIKNYWRTRVQKH

>mrna05167.1v1.0hybrid

RGRWTAEEDEILTNYIQLHGEGSWRSLPKNAGLLRCGKSCRLRWINYLRTDLRRGNITQEEEETIVKLHTALGNRWSLIAAQLPGRTDNEIKNYWNSHLSRK

>mrna07416.1v1.0hybrid

RGAWTAMEDRTLTEYITTHGEGKWRNLPKRAGLKRCGKSCRLRWLNYLRPDIKRGNITRDEEELIIRLHKLLGNRWSLIAGRLPGRTDNEIKNYWNTNIGKK

>mrna07418.1v1.0hybrid

KGAWTAEEDQKLAEVIAIHGAKKWKSIAAKAGLNRCGKSCRLRWLNYLRPNIKRGNISDQEEDLILRLHKLLGNRWSLIAGRLPGRTDNEIKNYWNSHLSKR

>mrna07646.1v1.0hybrid

KGAWTKEEDDRLIAYIRAHGEGCWRSLPKAAGLLRCGKSCRLRWINYLRPDLKRGNFTEEEDELIIKLHSLLGNKWSLIAGRLPGRTDNEIKNYWNTHIRRK

>mrna08084.1v1.0hybrid

KGPWTPEEDHVLVSYIQQYGHGNWRALPKLAGLLRCGKSCRLRWTNYLRPDIKRGNFSREEEEAIINLHQMLGNRWSAIAARLPGRTDNEIKNVWHTHLKKK

>mrna08535.1v1.0hybrid

RQRWRPEEDALLRAYVKQYGPREWNLVSQRMSTPLNRDAKSCLERWKNYLKPGIKKGSLTEEEQRLVICLQEKHGNKWKKIAAEVPGRTAKRLGKWWEVFKEKQ

>mrna08793.1v1.0hybrid

KGPWSKEEDEIIVELVEKYGPKKWSTIAQHLPGRIGKQCRERWHNHLNPGINKEAWTQEEELALIRAHQIYGNKWAELTKFLPGRTDNAIKNHWNSSVKKK

>mrna09039.1v1.0hybrid

KGPWSAEEDRVLTRLVDRYGPRNWSLISRYIKGRSGKSCRLRWCNQLSPTVQHRPFSQAEDEAILAAHARYGNRWATIARLLPGRTDNAVKNHWNSTLKRR

>mrna09311.1v1.0hybrid

RGHWRPAEDEKLRELVERYGPHNWNAIAEKLQGRSGKSCRLRWFNQLDPRINRNPFSEEEEERLLASHRIHGNRWAVIARLFPGRTDNAVKNHWHVIMARR

>mrna09406.1v1.0hybrid

KGAWSKQEDEKLTQYIQKHGEGSWRSLPVAAGLLRCGKSCRLRWVNYLRPDLKRGNFGEDEEDLIIKLHALLGNRWSLIAGRLPGRTDNEVKNYWNTHLRRK

>mrna09407.1v1.0hybrid

KGAWSIQEDQKLIDYIQKHGEGCWNSLPKAAGLRRCGKSCRLRWINYLRPDLKRGSFSEDEEDLIIRLHKLLGNRWSLIAGRLPGRTDNEVKNYWNSHLKKK

>mrna09845.1v1.0hybrid

KGLWSPEEDEKLFNYITRFGVGCWSSVPKLAGLQRCGKSCRLRWINYLRPDLKRGMFSQQEEDLIISLHEVLGNRWAQIAAQLPGRTDNEIKNFWNSCLKKK

>mrna10329.1v1.0hybrid

RQRWQPEEDALLRAYVKQYGPREWALVSQRMGQPINRDPKSCLERWKNYLRPGLKKGSLSPEEQSLVIELQAKYGNKWKKIAAELPGRTPKRLGKWWEVFRDKQ

>mrna11462.1v1.0hybrid

KGPWTTEEDQILISYIQTYGHGNWRALPKHAGLLRCGKSCRLRWINYLRPDIKRGNFSREEEDAIINLHEMLGNRWSAIAARLPGRTDNEIKNVWHTHLKKR

>mrna12738.1v1.0hybrid

KGAWTALEDRVLSEYIKVHGEGGWRNLPKKAGLKRCGKSCRLRWLNYLRPDIKRGNISPDEEELIVRLHKLLGNRWSLIAGRLPGRTDNEIKNYWNTNLSKR

>mrna14666.1v1.0hybrid

KGLWSPEEDEKLMRYMLNNGQGCWSDVARNAGLERCGKSCRLRWINYLRPDLKRGAFSPQEEDLIIHFHSLLGNRWSQIAARLPGRTDNEIKNFWNSTIKKR

>mrna15383.1v1.0hybrid

RGSWTAEEDQRLAQVIEVYGPRKWKSVATKAGLNRCGKSCRLRWMNYLRPNIKRGNISDQEEDLILRLHKLLGNRWSLIAGRLPGRTDNEIKNYWNSHLSKK

>mrna15386.1v1.0hybrid

KGAWTAHEDQVLRDYVKENGEGKWGKMSRETGLKRCGKSCRLRWLNYLRPDIKRGNITEDEEELIIRLHKLLGNRWSLIAGRLPGRTDNEIKNYWNSTLRKK

>mrna15392.1v1.0hybrid

RGAWTALEDKVLTSYIKAHGEGKWRNLPKRAGLKRCGKSCRLRWLNYLRPDIKRGNISGDEEELIIRLHNLLGNRWSLIAGRLPGRTDNEIKNYWNTTLAKK

>mrna16287.1v1.0hybrid

KGPWTPEEDQKLMNYIQKHGHGSWRALPKLAGLNRCGKSCRLRWTNYLRPDIKRGKFSQEEEQTILNLHSILGNKWSAIASHLPGRTDNEIKNFWNTHLKKK

>mrna18416.1v1.0hybrid

KGPWTQEEDDKIVELVAKYGPTKWSLISKSLPGRIGKQCRERWHNHLNPDIKREAWTLVEELALMHAHRMHGNKWAEIAKALPGRTDNAIKNHWNSSLKKK

>mrna18421.1v1.0hybrid

RQRWKPEEDALLRAYVKQYGPREWNLVSQRMSTPLNRDAKSCLERWKNYLKPGIKKGSLTEEEQRLVICLQEKHGNKWKKIAAEVPGRTAKRLGKWWEVFKDKQ

>mrna18691.1v1.0hybrid

RGPWTPREDTLLTKYIEAHGEGHWRSLPKKAGLLRCGKSCRLRWMNYLRPDIKRGNITPDEDDLIIRLHSLLGNRWSLIAGRLPGRTDNEIKNYWNTHLSKR

>mrna18756.1v1.0hybrid

RGVWSQEEDRILSRLVSKFGARNWTLIARGIPGRCGKSCRLRWFNQLDPILKRKPFSAEEDRMIISAHAIHGNKWSAIAKLLPGRTDNAIKNHWNSTLRRR

>mrna19065.1v1.0hybrid

RGPWTPDEDEILVNYINKNKGHGSWRSLPQHAGLLRCGKSCRLRWTNYLRPDIKRGPFTEEEEKLIIQLHGMLGNRWAAIASQLPGRTDNEIKNLWNTHLRKR

>mrna19115.1v1.0hybrid

KGFWSKEEDDLIIELVAKQGNKKWSEIAKSLPGRIGKQCRERWHNHLNPDIKRTAWTNEEEQILIQSHKVYGNKWAEIAKFLPGRTENSIKNHWNCSVKKK

>mrna20888.1v1.0hybrid

RGSWSPKEDELLTRLVTQFGARNWSVIARGIPGRSGKSCRLRWCNQLNPGVKRKPFSDEEDRIIVAAHATHGNRWSIIAKLLPGRTDNAIKNHWNSTLKRG

>mrna21001.1v1.0hybrid

KGSWSPQEDATLIKLVAEHGPRNWSLISSGIPGRSGKSCRLRWCNQLSPDVQHKPFTPAEDAAIVTAHALHGNKWATIARLLPGRTDNAIKNHWNSTLRR

>mrna22961.1v1.0hybrid

KGLWSPEEDEKLLNYITKHGHGCWSSVPKLAGLQRCGKSCRLRWINYLRPDLKRGPFSQQEENLIIELHAVLGNRWSQIAAQLPGRTDNEIKNLWNSCIKKK

>mrna24027.1v1.0hybrid

KGPWSPEEDDALQSLVQKHGPRNWSLISKAIPGRSGKSCRLRWCNQLSPQVEHRAFTPEEDDTIIRAHARFGNKWATIARLLSGRTDNAIKNHWNSTLKRK

>mrna25098.1v1.0hybrid

RGPWTRREDTLLIQYIQSHGEGHWKSVPKKAGLLRCGKSCRLRWINYLRPDIKRGNITPDEEDLIARLHSLLGNRWSLIAGRLPGRTDNEIKNYWNTKLSKR

>mrna25149.1v1.0hybrid

RGPWTLEEDTLLIQYIARHGEGRWNLLANRAGLRRTGKSCRLRWLNYLKPDVKRGNLTPEEQLMILDLHSKWGNRWSKIAQYLPGRTDNEIKNYWRTRVQKQ

>mrna25685.1v1.0hybrid

KGAWTKEEDQRLIDYIRIHGEGCWRSLPKQAGLLRCGKSCRLRWINYLRPDLKRGNFTEEEDELIIKLHSLLGNKWSLIAGRLPGRTDNEIKNYWNTHIKRK

>mrna26007.1v1.0hybrid

KGPWTPEEDIILVSYIQEHGPGNWRSVPTNTGLMRCSKSCRLRWTNYLRPGIKRGNFTDHEEKMIIHLQALLGNRWAAIASYLPQRTDNDIKNYWNTHLKKK

>mrna26044.1v1.0hybrid

RGPWTPQEDKKLFAFIQQHGHGSWRSLPEKAGLQRCGKSCRLRWKNYLRPDIKRGNFSLQEDQTIIQLHALLGNRWSAIAANLPRRTDNEIKNYWNTHLKKR

>mrna26045.1v1.0hybrid

RGQWTPEEDEKLFSFVQKHGHGSWRSLPRKAGLQRCGKSCRLRWKNYLNPDIKRGNFSLQEDQTIIQLHALLGNRWSAIAAQLPKRTDNEVKNYWNTHLKKR

>mrna26289.1v1.0hybrid

KGPWSPEEDDLLRKLVQRHGARNWTLISKSIPGRSGKSCRLRWCNQLSPEVEHRAFTAEEDEIIAAAHSKYGNKWATIARLLNGRTDNAIKNHWNSTLKRK

>mrna27001.1v1.0hybrid

KGLWSPEEDEKLMRYMLSNGQGCWSDIARNSGLQRCGKSCRLRWINYLRPDLKRGAFSPQEEHLIIHFHSILGNKWSQIAARLPGRTDNEIKNFWNSALKKR

>mrna28293.1v1.0hybrid

KGPWTSEEDRLLIEYVRLHGEGRWNSVARLAGLKRNGKSCRLRWVNYLRPDLKRGQITPHEESIILQLHARWGNRWSTIARSLPGRTDNEIKNYWRTHFKKK

>mrna28435.1v1.0hybrid

KGPWTMEEDLILINYIANHGEGVWNSLAKSAGLKRTGKSCRLRWLNYLRPDVRRGNITPEEQLLIMELHAKWGNRWSKIAKHLPGRTDNEIKNYWRTRIQKH

>mrna29254.1v1.0hybrid

RGHWRPAEDSKLKQLVAQFGPQNWNLIAEHLDGRSGKSCRLRWFNQLDPRINRRAFSEEEEERLLSAHRLYGNKWAMIARLFPGRTDNAVKNHWHVIMARK

>mrna29961.1v1.0hybrid

RGPWSPSEDLRLISFIQKHGHDNWRALPKQAGLLRCGKSCRLRWINYLRPDLKRGNFTKEEEESIIMLHEAWGNKWSKIASHFPGRTDNEIKNVWNTHLRKK

>mrna30024.1v1.0hybrid

KGPWTPEEDQKLVQYIQEHGHGSWRALPKLAGLNRCGKSCRLRWTNYLRPDIKRGNFSQEEEQTILHLHSLLGNKWSAIATHLSGRTDNEIKNFWNTHLKKK

>mrna30725.1v1.0hybrid

KGPWTPEEDQKLVDYIQKHGYGNWRTLPKNAGLQRCGKSCRLRWTNYLRPDIKRGRFSFEEEETIVQLHSILGNKWSAIAARLPGRTDNEIKNYWNTHIRKR

>mrna31413.1v1.0hybrid

KGASTKEEDELLKQFIEIHGEGKWHHVPLKSGLNRCRKSCRLRWLNYLKPNIKRGEFAEDEVDLIIRLHKLLGNRWSLIAGRLPGRTANDVKNYWNTYQRKK

>mrna31835.1v1.0hybrid

RGLWSPEEDEKLIRYITTHGYGCWSEVPEKAGLQRCGKSCRLRWINYLRPDIRRGRFTPEEEKLIISLHGVVGNRWAHIASHLPGRTDNEIKNYWNSWIKKK

>TRINITY_DN32830_c1_g2_i1

KGPWSPEEDEALQRLVQSYGPRNWSLISKSIPGRSGKSCRLRWCNQLSPEVEHRPFTPEEDDTIIRAHARFGNKWATISRLLNGRTDNAIKNHWNSTLKRK

>TRINITY_DN40669_c1_g2_i2

KGPWSPEEDDALQSLVQKHGPRNWSLISKAIPGRSGKSCRLRWCNQLSPQVEHRAFTPEEDDTIIRAHARFGNKWATIARLLNGRTDNAIKNHWNSTLKRK

>TRINITY_DN32923_c0_g1_i1

RGPWSPEEDKVLSRLVSKFGARNWSLIARGISGRSGKSCRLRWCNQLDPILKRKPFSEEEDQMIIVAHTIHGNKWAAIARLLPGRTDNAIKNHWNSTLRRR

>TRINITY_DN30644_c0_g1_i1

RGHWRPAEDEKLRELVERYGPHNWNAIAEKLQGRSGKSCRLRWFNQLDPRINRNPFSEEEEERLLASHRIHGNRWAVIARLFPGRTDNAVKNHWHVIMARR

>TRINITY_DN40785_c0_g1_i4

RQRWRPEEDALLRAYVKQYGPREWNLVSQRMNTPLNRDAKSCLERWKNYLKPGIKKGSLTEEEQRLVICLQEKHGNKWKKIAAEVPGRTAKRLGKWWEVFKEKQ

>TRINITY_DN12034_c0_g3_i1

KGPWTPEEDHVLVSYIQQYGHGNWRALPKLAGLLRCGKSCRLRWTNYLRPDIKRGNFRREEEEAIINLHQMLGNRWSAIAARLPGRTDNEIKNVWHTHLKKK

>TRINITY_DN28996_c0_g1_i1

KGPWTPEEDIILVSYIQEHGPGNWRSVPTNTGLMRCSKSCRLRWTNYLRPGIKRGNFTDHEEKMIIHLQALLGNRWAAIASYLPQRTDNDIKNYWNTHLKKK

>TRINITY_DN32810_c2_g2_i1

KGPWTPEEDQKLLAYIEEHGHGSWRALPIKAGLQRCGKSCRLRWTNYLRPDIKRGKFSLQEEQTIIQLHALLGNRWSAIATHLPKRTDNEIKNYWNTHLKKR

>TRINITY_DN34526_c0_g1_i1

KGLWSPEEDEKLLNYITKHGHGCWSSVPKLAGLQRCGKSCRLRWINYLRPDLKRGPFSQQEENLIIELHAVLGNRWSQIAAQLPGRTDNEIKNLWNSCIKKK

>TRINITY_DN15240_c0_g3_i1

KGAWTAHEDQVLRDYVKEYGEGKWGKMSRETGLKRCGKSCRLRWLNYLRPDIKRGNITEDEEELIIRLHKLLGNRWSLIAGRLPGRTDNEIKNYWNSTIRKK

>TRINITY_DN33327_c0_g2_i2

RGPWTPREDTLLTKYIEAHGEGHWRSLPKKAGLLRCGKSCRLRWMNYLRPDIKRGNITPDEDDLIIRLHSLLGNRWSLIAGRLPGRTDNEIKNYWNTHLSKR

>TRINITY_DN26257_c0_g1_i1

RGAWTALEDKVLTSYIKAHGEGKWRNLPKRAGLKRCGKSCRLRWLNYLRPDIKRGNISGDEEELIIRLHNLLGNRWSLIAGRLPGRTDNEIKNYWNTTLVKK

>TRINITY_DN36867_c3_g2_i1

KGAWTKEEDQRLIDYIRIHGEGCWRSLPKQAGLLRCGKSCRLRWINYLRPDLKRGNFTEEEDELIIKLHSLLGNKWSLIAGRLPGRTDNEIKNYWNTHIKRK

>TRINITY_DN23499_c0_g1_i1

RGRWTAEEDEILTNYIQLHGEGSWRSLPKNAGLLRCGKSCRLRWINYLRTDLRRGNITQEEEETIVKLHTALGNRWSLIAAHLPGRTDNEIKNYWNSHLSRK

>TRINITY_DN26493_c0_g2_i3

RGPWTVEEDLALMNYIANHGEGRWNSLARCAGLKRTGKSCRLRWLNYLRPDVRRGNITLEEQLLILELHSRWGNRWSKIAQHLPGRTDNEIKNYWRTRVQKH

>TRINITY_DN39667_c0_g1_i2

KGPWTLDEDTLLIHYIANHGEGHWNALAKCAGLKRTGKSCRLRWLNYLKPDIKRGNLTPQEQLLILELHAKWGNRWSKIAQHLPGRTDNEIKNYWRTRVQKQ

>TRINITY_DN29300_c0_g1_i3

KGAWTKEEDELLRQVIEKHGEGKWHQVPFKAGLNRCRKSCRLRWLNYLKPNIKRGEFTVDEVDMIIRLHKLLGNRWSLIAGRLPGRTANDVKNYWNTYQRKK

>TRINITY_DN44524_c0_g1_i1

RGAWTAMEDRILREYITTHGEGKWRNLPKRAGLKRCGKSCRLRWLNYLRPDIKRGNITRDEEELIIRLHKLLGNRWSLIAGRLPGRTDNEIKNYWNTNIGKK
